# The *Drosophila foraging* Gene Mediates Adult Plasticity and Gene–Environment Interactions in Behaviour, Metabolites, and Gene Expression in Response to Food Deprivation

**DOI:** 10.1371/journal.pgen.1000609

**Published:** 2009-08-21

**Authors:** Clement F. Kent, Tim Daskalchuk, Lisa Cook, Marla B. Sokolowski, Ralph J. Greenspan

**Affiliations:** 1 Department of Biology, University of Toronto Mississauga, Ontario, Canada; 2 Phenomenome Discoveries, Saskatoon, Saskatchewan, Canada; 3 The Neurosciences Institute, San Diego, California, United States of America; The University of Queensland, Australia

## Abstract

Nutrition is known to interact with genotype in human metabolic syndromes, obesity, and diabetes, and also in *Drosophila* metabolism. Plasticity in metabolic responses, such as changes in body fat or blood sugar in response to changes in dietary alterations, may also be affected by genotype. Here we show that variants of the *foraging* (*for*) gene in *Drosophila melanogaster* affect the response to food deprivation in a large suite of adult phenotypes by measuring gene by environment interactions (GEI) in a suite of food-related traits. *for* affects body fat, carbohydrates, food-leaving behavior, metabolite, and gene expression levels in response to food deprivation. This results in broad patterns of metabolic, genomic, and behavioral gene by environment interactions (GEI), in part by interaction with the insulin signaling pathway. Our results show that a single gene that varies in nature can have far reaching effects on behavior and metabolism by acting through multiple other genes and pathways.

## Author Summary

Normal individual differences in the *foraging* (*for*) gene of the fruit fly *Drosophila melanogaster* result in two behavioral types called rover and sitter. Larval rovers show a greater behavioral response to changes in their food environment than sitters. The *for* gene makes an enzyme called PKG, which is found in the head of the fly, as well as in most other organisms, including humans. Here, we demonstrate that adult rovers and sitters differ in their metabolic response to changes in their food environment. We measure metabolites in rovers and sitters and show that rovers store energy predominantly as lipids, whereas sitters store it as carbohydrates. We also examine expression levels of genes in rover and sitter heads when the flies are well-fed or food-deprived. We find that *for* affects levels of gene products involved in carbohydrate and fat metabolism and insulin signaling. We confirm an interaction between *for* and insulin signaling genes by using genetic mutants to measure their combined effects on fly food-leaving behavior. Our results show that natural variation in this single gene can affect plasticity of large numbers of traits.

## Introduction

The question of how phenotypic plasticity evolves has been the subject of vigorous debate (reviewed in [1],[2],[3]), as has the related question of whether allelic variation in single genes can have large impacts on plasticity [3]. Phenotypic plasticity is defined as the degree to which the environment can change or modify the phenotype. Genotype-environment interaction (GEI) is genetic variation in phenotypic plasticity. The genetic variation in GEI is needed for the evolution of an adaptive level of phenotypic plasticity [4]. Here we abbreviate “the phenotypic plasticity of one genotype in different environments” to “plasticity”. Recent studies show that quantitative trait loci can have large effects on GEI [5] and that traits with GEI responses to nutrition can be correlated with GEI in the expression of a relatively small number of genes [6].

In this paper, we examine GEIs resulting from variation in a single gene, in response to food deprivation. We begin the process of determining the mechanism by which alleles of this gene affect plasticity. By quantifying the proportion of a large number of gene expression and metabolite traits in which the gene is involved in GEI, we also provide experimental data on the extent of the gene's pleiotropy and its allelic contributions to plasticity.

The *foraging* (*for*) gene of *D. melanogaster* encodes a cGMP dependent protein kinase [7],[8]. Naturally occurring *for* alleles give rise to the rover and sitter behavioural morphs. As larvae, rovers move more and feed less in the presence of food than sitters, but don't differ in locomotion in the absence of food [9],[10],[11]. Food deprivation causes larval rovers to behave more like sitters [12]. Like their larval counterparts, adult rovers and sitters also differ in food-related behaviours. The sucrose response of rovers in a proboscis extension assay is higher than in sitters and the patterns of walking after feeding on a sucrose drop in sitters exhibits higher turning rates than in rovers [13],[14],[15].

In the present study, we investigate the response of adult rovers and sitters to well-fed (Fed) and food deprived (FD) conditions. Through global profiling of gene expression and metabolites, we find that rovers have greater changes in gene expression profiles and metabolite levels in response to food deprivation than do sitters, and that the insulin pathway is required for this rover-sitter difference. Allelic variation in *for* also influences the allocation of energy stores to lipids as compared to carbohydrates in fed flies. We conclude that allelic variation in *for* has a major effect on multiple aspects of food-related plasticity and GEI.

## Results

We performed behavioural assays and gene expression profiling on flies from natural rover (*for^R^*) and sitter (*for^s^*) strains and from a sitter mutant strain (*for^s2^*) generated on a rover (*for^R^*) genetic background [9],[13]. We exposed these flies to well-fed (Fed) or food deprived conditions (FD) (see methods). To contain cost, metabolite profiling was limited to the *for^R^* and *for^s2^* strains.

For behaviour, metabolite and gene expression measures we calculate *I*, the difference between rover and sitter plasticity, as *I* = (fed rovers – FD rovers)−(fed sitters – FD sitters); that is, *I* is the difference between rover response to food and sitter response (for cases when two sitter strains are used, see Statistical Methods). *I* can be measured for behavioural, metabolic, and gene expression traits, and gives useful information about GEI. *I* is proportional to the Analysis of Variance (ANOVA) interaction term used to calculate the significance of GEI. Thus, the “direction of GEI”, refers to the sign of *I*.

### Behaviour

**Figure 1:**
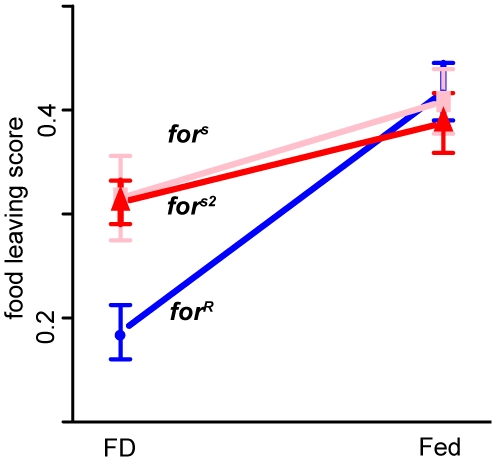
*foraging* gene by food interaction in behaviour. The interaction between *for* and food [Fed vs. food deprived (FD)] is significant (p = 0.021) and positive in sign (rovers increase more than sitters; *I*>0) (for medium composition and ANOVA results see Table S1A, S1B, and Methods). The food-leaving score (arcsine-transformed proportion of flies leaving a known food source and traversing a maze; see Methods for assay details) is plotted±1 standard error of the mean.

The food-leaving assay measures the proportion of flies that traverse a maze after leaving a vial containing sucrose in agar (see Methods and Figure S1). This “food-leaving” behaviour shows significant GEI interaction (Figure 1). To determine how general this pattern of GEI is we repeated these experiments rearing flies on a variety of different food media and in all cases we found significant and similar patterns in GEI (Table S1 for descriptions of food media and statistical tables). Specifically, rover scores increase more from FD to Fed environments than sitter scores. The direction of the GEI is positive (*I*>0) in all cases.

### Metabolites

**Figure 2:**
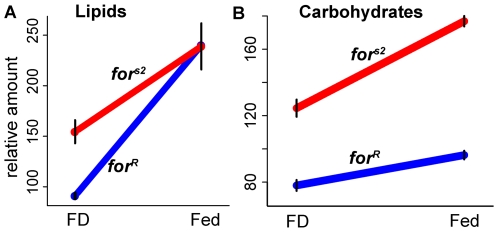
Rovers and sitters use energy stores differently. Change in the heads of flies in (A) triacylglycerols (TAG) and (B) polysaccharides (PS) between Fed and food deprived (FD) states is higher in rovers than in mutant sitters (positive GEI interaction *I*>0) for TAG but lower for PS (negative GxE interaction *I*<0; both interactions significant, Table S2A has ANOVA details). Total signal/noise ratio levels determined using FTICR shown on vertical axis (Methods). FTICR measurements were done on rovers (*for* ^R^) and mutant sitters (*for* ^s2^).

We examined Fed and FD *for^R^* and *for^s2^* heads to determine compounds most strongly associated with the *for* response to feeding state, using Fourier Transform Ion Cyclotron Resonance Mass Spectroscopy (FTICR MS, Methods) to detect 750 putative metabolites. We found a significant influence of *for* (*for^R^* or *for^s2^*) and feeding state on compounds with the molecular weight (MW) and chemical properties of triacylglycerols (TAG) and polysaccharides (PS) (Figure 2A and 2B; Table S2A, S2B). There was a significant main effect of *for* in carbohydrates, and significant GEI in both carbohydrates and lipids, but in opposite directions (*I*>0 for lipids, *I*<0 for carbohydrates). The largest differences were found in smaller MW compounds. Incorporating MW into ANOVA of TAG compounds gave p(*for*×food) = 1.3·10^−13^ and p(*for*×MW) = 1.9·10^−5^; PS compounds had p(*for*×food×MW) = 0.03 (Table S2B). (Note that the term food in the ANOVA describes the feeding state of Fed vs. FD and the term *for* refers to the strains *for^R^* or *for^s2^*). Thus, GEI interactions are found for metabolites but their direction *I* depends on metabolite type. Rovers have a larger drop in lipids than sitters in the change from Fed to FD, while sitters have a greater drop in carbohydrates than rovers.

**Figure 3:**
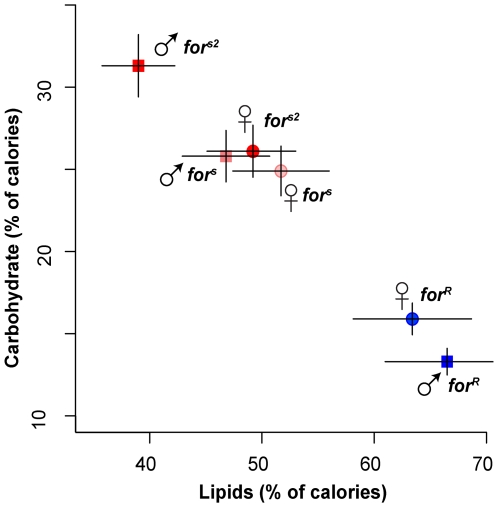
Fed rovers and sitters store energy differently. The proportion of total calories due to lipids (horizontal axis) and carbohydrates are shown in whole-body measurements of fed 5-day-old males and females for the two sitter (red) and one rover (blue) strains. Data are standardized for fly dry weight (Methods). Rovers store significantly more energy as lipids and significantly less as carbohydrates. For males and for females, mutant and natural sitters didn't differ (Welch's t-test, p>.17 all tests) and hence were pooled for a rovers versus sitters comparison. Lipids: males, t = 3.26, df = 3.41, p = 0.039; females t = 5.08, df = 10.2, p = 0.0004. Carbohydrates: males, t = −3.98, df = 9.20, p = 0.003; females t = −5.64, df = 7.84, p = 0.0005. Data for *n* = 5 except *n* = 4 for male lipids. Error bars represent ±1 s.e.m.

Whole-fly spectrophotometric measures of total carbohydrates, lipids, and proteins (Methods) showed that adult rovers had almost twice as much energy stored in whole-body lipid and about half the energy stored in carbohydrates compared to adult sitters, whereas protein levels normalized to dry weight were not significantly different between genotypes (Figure 3 gives full statistics). Thus, *for* genotype strongly affects energy storage strategies. A main effect of genotype in fed flies is consistent with an allocation shift between storage of energy as lipids and as carbohydrates.

### GEI in gene expression depends on metabolic role

If *for* affects both behavioural and metabolic GEI and plasticity in a food-dependent manner, how is this reflected at the level of gene expression? To examine *for*'s effect on transcript levels we performed whole-genome microarray analysis on heads of rovers and sitters and sitter mutants under Fed and FD conditions (Methods). Array results were verified using qRTPCR on two genes with strong rover-sitter differences and involved in carbohydrate metabolism [*Treh*, trehalase, and *CG10924*, human homolog is *PCK1* phosphoenolpyruvate carboxykinase 1 (soluble)] (Figure S3).

**Figure 4:**
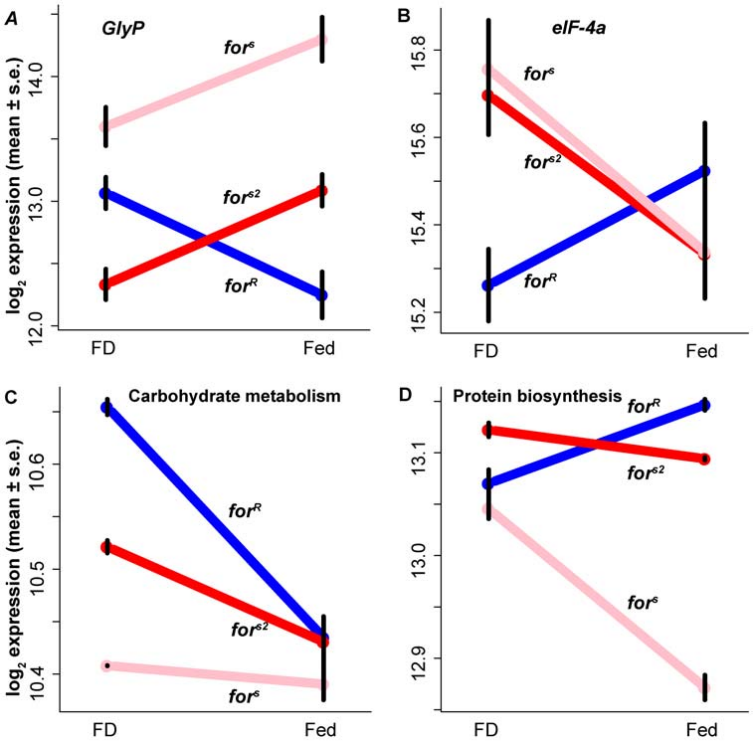
Transcriptional interactions between *foraging* alleles and food. Individual genes involved in energy metabolism have significant *for*×food interactions (Int) with GEI *I*<0 for catabolism and *I*>0 for anabolism (top row), while two functional groups of genes involved in catabolism and anabolism have group-level interactions in the same pattern (bottom row). (A) Glycogen phosphorylase (*GlyP*), a key enzyme in glycogen breakdown; q(Int) = 5.68×10^−5^, *I*<0. (B) eukaryotic translation initiation factor 4A (*eIF-4A*), part of the translation initiation complex; q(Int) = 0.0041, *I*>0. (C) Group-level ANOVA (see Methods) for Gene Ontology (GO) group 5975, carbohydrate metabolism; q(Int) = 0.032, *I*<0. (D) GO group 6412, protein biosynthesis; q(Int) = 0.0023, *I*>0 (Table S3 has GO group ANOVA tables). Error bars are ±1 s.e.m. There is a significant background (BG)×food interaction in (d) (q = 8.75×10^−7^); for (A–C) there is no BG×food interaction; there is a BG main effect in (A,C,D).

Overall, the expression of genes involved in the breakdown of food to provide energy (catabolism) was significantly altered (had strong GEI), with rovers decreasing and sitters increasing their expression when food is present (*I*<0). For instance, glycogen phosphorylase (*GlyP*), which regulates glycogen breakdown, is highly significant (Figure 4A, q(*for*×food) = 0.000057). The differing genetic background between *for^s^* and *for^s2^* has a main effect on *GlyP* (q(BG) = 1.64·10^−7^), but the response to food of each sitter genotype is similar (q(BG×food) = 0.33), so it is meaningful to speak of a GEI common to both types of sitters when compared to rovers. Conversely, expression of many genes in pathways for synthesis of proteins (anabolism) significantly increased in rovers in the Fed condition, exemplified by eukaryotic translation initiation factor 4A (*eIF-4A*; Figure 4B, q(*for*×food) = 0.0011). *I* is positive for *eIF-4A* and negative for *GlyP*.

We investigated whether functionally related genes show common modes of regulation as defined by the sign of *I* (see Text S1, Supplementary Methods, Group Level ANOVA), and found effects consistent with the catabolism-anabolism pattern described above. Catabolic groups such as carbohydrate (Figure 4C), glycogen, chitin, and amino acid breakdown genes, and mitochondrial oxidative phosphorylation complexes I–V had significant negative *I* values (Table S3). Anabolic groups associated with protein biosynthesis (Figure 4D), such as gene splicing, translation initiation, ribosomal proteins, and post-translational protein modification complex had significant positive *I* values (Figure 4D, Table S3). In Table S3, we list Gene Ontology groups with significant GEI, ordered from most positive to most negative *I* [groups with greater GO term specificity as per DAVID [16] GO levels 3–5 were used]. The most inclusive GO groups relating to catabolism/anabolism are group 9056 “catabolic process” which has significant negative *for*×food interaction *I* (*p* = 0.009, n = 140 genes) while the “biosynthetic process” group 9058 has positive *I* (*p* = 0.008, n = 300 genes; as these two groups were specifically singled out for testing we report *p* rather than FDR-adjusted *q*).

Some gene groups with significant *for* GEI go beyond a simple anabolism - catabolism dichotomy. In particular, functional groups involved in neural and muscle function – neurotransmitter secretion groups, synaptic transmission, postsynaptic membrane, ion channels, GABA and calcium-binding EGF domains – all have negative *I* values (Table S3).

### Importance of genetic background in gene expression

How does the genetic background (BG) difference between *for^s^* and the other two strains (*for^R^* and *for^s2^*) affect GEI of functional groups? Most groups in Table S3 have highly significant FDR-corrected q values for the main effect of background. However, only a few groups have significant BG×food interactions, and these are all groups with positive *for*×food *I* values, associated with transcription, splicing, translation, or post-translational modification of proteins. Thus, at the functional group level, gene groups with significant positive *for* GEI *I* often have significant interactions with the genetic background but those with negative *I* values do not.

We used two methods to quantify the contributions of *for*, food, and BG to gene expression over all genes above cut off, not just those with interactions. First, we performed a principal components analysis (PCA) on log_2_ gene expression levels. Five PCA components were identified. The first component (explaining the largest amount of variance, see below) correlates strongly with main effect of BG, followed by components correlating with main effects of food and then *for*, with components 4 and 5 (explaining similar amounts of variance) correlating with the interactions of BG×food and *for*×food. Thus, genetic effects rank in the order BG>*for*>BG×food>*for*×food when measured by variance explained.

Our second method uses the Storey-Tibshirani false discovery rate (FDR) analysis to estimate the proportion of genes with significant effects [17]. This method uses a mixture-model approach to estimate the proportion π_0_ of genes matching the null hypothesis of no effect. Then π_alt_ = 1−π_0_ estimates the proportion of genes matching the alternative hypothesis of an effect. One interpretation of π_alt_ is that is the proportion of genes that would show significant effects, after FDR, if we had large numbers of replicates. π_0_ and hence π_alt_ depend not only on the true rate of differential expression of genes (DEG) but also on the signal to noise ratio of the array technology. Thus, for low expression genes with poorer signal to noise ratio π_0_ will be higher even if the true proportion of DEG is unchanged. We calculated π_0_ both for the top-1000 highest expressing genes and for increasingly large groups of genes based on minimum expression levels. If signal to noise is the only factor affecting π_0_ then the intercept of the curve of π_0_ values based on expression is an estimator of 1-DEG. In practice we found good agreement between the latter method and π_0_ for the top 1000 genes, so we report the top-1000 figure.

This analysis was done for each ANOVA p-value (for all main and interaction effects). The π_alt_ = 1−π_0_ for a given set of p-values (e.g. *for* main effect p values) estimates the true DEG rate for that effect. Our top-1000 π_alt_ values were BG = 0.84, food = 0.73, BG×food = 0.63, *for* = 0.59, and *for*×food = 0.57. That is, among the top-1000 genes by mean expression level, 84% had a main effect of BG, 73% of food, 63% showed BG×food, 59% had a main effect of *for* and 57% had *for*×food GEI. Thus, although *for*×food GEI affects the smallest proportion of top-expressing genes, it still has an effect on 57% of these genes.

### A quantitative measure of plasticity is higher in rovers

The interaction between *for* and feeding state could be due either to the genotypes responding in equal amounts but opposite directions to feeding (same plasticity of each genotype), or to one genotype responding more than the other (differences in magnitude of plasticity). To quantify differences in plasticity between rovers and sitters, we calculated an index of plasticity called Relative Nutrient Sensitivity (RNS) for any given trait as the difference between the size of the trait's rover response to food and the size of the sitter response: RNS = (|rover change|−|sitter change|)/C (where C = 1 for log_2_ transformed data, otherwise C = mean level of trait). In other words, for each trait compared (e.g., behaviour, metabolite, gene expression), RNS compares the absolute magnitude of *for*-dependent changes in response to food rather than their direction (see Methods).

**Figure 5:**
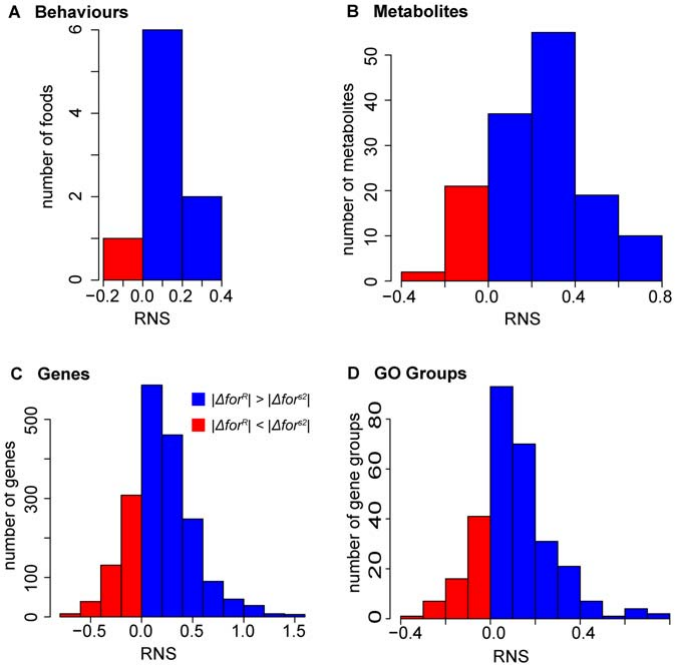
*foraging* GEI is due to plasticity differences: rovers respond more to food than sitters. In each histogram, the horizontal axis is the measure RNS (relative nutrient sensitivity; Methods) of which genotype has larger response to food. Blue bars, rovers respond more (RNS>0); red bars, mutant sitters respond more. (A) Behavioural plasticity: RNS measured using 9 different food media (Table S1C). RNS>0 for 8 of 9 (89%) and RNS = −0.004 for the ninth. Student t for RNS≠0, t = 2.99, df = 8, p = 0.009. (B) Metabolite plasticity: RNS for compounds with a significant response to food. 84% of these had RNS>0. Chi-square contingency test χ^2^ = 65.3, df = 1, p = 6.3×10^−16^. (C) Gene expression plasticity: RNS for 1,000 genes with significant food response. Of these, 77% had RNS>0 (χ^2^ = 305.3,df = 1, p<2.2×10^−16^). (D) Functional group plasticity. RNS for 300 Gene Ontology groups with significant food response. In 77% of these RNS>0 (χ^2^ = 88.6,df = 1, p<2.2×10^−16^). Average mutant sitter change on food deprivation is about ½ of rover. For simplicity, only rover versus mutant sitter RNS values are shown. This is conservative; rover versus natural sitter gene and gene group RNS distributions were more biased in favour of rovers than the rover vs. mutant sitter (Kolmogorov-Smirnov test for genes, D = 0.236, p<2.2×10^−16^; for gene groups D = 0.233, p = 0.000022).

When RNS>0, rovers show a larger response; when RNS<0, sitters respond more. When we tabulated RNS for behaviour, metabolites, gene expression, and functional gene categories, we found RNS>0 (rovers change more) in 8 of 9 behaviour cases (89%, Figure 5A, p = 0.03). For metabolites, gene expression, and functional gene categories, a significant majority of traits had RNS>0 (Figure 5B–5D, p<10^−15^ and Table S4). Thus, for a large majority of behavioural, metabolic, and gene expression traits, rovers exhibit greater food-related plasticity than sitters. This is true whether *for^s2^* or *for^s^* are used in comparison to *for^R^*. The rover-natural sitter RNS comparison is more rover-biased (has more cases with rovers changing more than sitters) than the rover-mutant sitter comparison, but the correlation between the two is good (for genes, *r* = 0.83, *p*<10^−15^), so we show the conservative, common genetic background, rover-sitter mutant distributions in Figure 5.

The direction of *for*-dependent response to feeding state (GEI *I*) and plasticity (RNS) are different measures, as shown in Table S3. Most groups with significantly negative GEI *I* have RNS>0. Of the traits with RNS<0, most are mitochondrial groups involved in fatty acid beta oxidation or oxidative phosphorylation complexes I–V. Among metabolites (Table S2A, Table S4) only the group containing PS polysaccharides has significant RNS<0. So, sitters are more plastic than rovers (have a higher magnitude of change in response to food) for a small subset of traits having to do with sugars (among metabolites) or mitochondrial catabolic pathways (functional groups), but rovers are more plastic for the majority of gene and metabolite groups.

### Hypothesis: plasticity differences and the insulin pathway

We hypothesized that the *for*-dependent metabolic plasticity might be mediated by the insulin signaling pathway. This is because rovers exhibit a higher plasticity in response to feeding state and the insulin signaling pathway is a key regulator of the response to food [18].

In the cell, binding of DILPS (Drosophila insulin-like peptides) to the insulin receptor (*InR*) triggers a signaling cascade with major effects on gene expression [19]. Protein translation is increased via phosphorylation of key members of the TOR pathway by the kinases *Akt1* (dPKB) and *Pk61C* (dPDK) [20],[21]. Negative homeostatic control of insulin signaling occurs on the transcriptional level – when signaling is high, *foxo* is phosphorylated by *Akt1* and sequestered in the cytoplasm, but when signaling drops *foxo* translocates to the nucleus where it stimulates transcription of genes such as *InR* and the negative regulator of translation *Thor (d4EBP)* [22],[23],[24]. At the transcript level, then, many insulin pathway genes have an inverse relationship to the level of insulin signaling. Our results show that transcription of positive regulators decreased more in fed rovers than sitters, resulting in a negative GEI interaction coefficient *I* for the group of positive regulators as a whole (Figure S2). This normal inverse relationship between transcription and insulin signaling is more evident in rovers than sitters. As with RNS, this is true whether mutant or natural sitters are compared, but again as for RNS, the difference between rovers and natural sitters is larger than the difference between rovers and mutant sitters, suggesting that the genetic background of natural sitters may intensify this difference.

### Genetic test of interaction of *foraging* and insulin genes

The finding that rovers show larger responses to food, and the known role of insulin signaling in the response to food, suggests that rovers might also show a larger impact of changes to insulin signaling. We therefore tested whether *for* interacts with the insulin signaling pathway by means of quantitative complementation crosses for epistasis between mutant insulin pathway genes and alleles of *for* [25],[26],[27]. We crossed each of the three *for* genotypes to loss of function mutants of the fly insulin receptor *InR*, phosphatidylinositol-3-kinase *Pi3K92E* (or *Dp110*), and *foxo* (Methods). *InR* and *Dp110* are positive regulators of insulin signaling; *foxo* is a negative regulator. Based on our gene expression data, we hypothesized that rovers had higher insulin signaling than sitters, so we expected crosses of rovers with loss of function insulin mutants to be more sitter-like than their controls.

**Figure 6:**
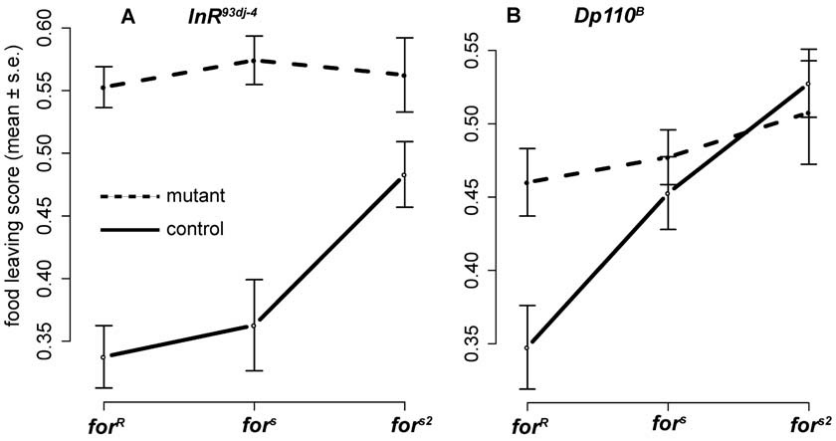
Insulin pathway genes interact with *foraging* alleles in expression and in food-leaving behaviour. Flies carrying different *for* alleles crossed to mutants of the positive regulators (A) *InR* and (B) *Dp110* (dashed curves) show almost none of the normal food-deprived rover-sitter food-leaving difference (compare to Figure 1 and solid balancer curves in this figure). In these quantitative complementation crosses, the food leaving behaviour of FD *for;mutant* transheterozygotes is compared to the FD *for;Balancer* transheterozygote controls. The difference in food leaving between the control balancer and mutant cross depends significantly on *foraging* allele, demonstrating interaction between the mutant gene and *for*. p(Interaction) = 0.012 (*InR*), p = 0.046 (*Dp110*). Data is arcsine transformed means±1 standard error for trials on *n* days (*n* = 11 for *InR* and *Dp110*). Behaviour assays were performed on FD flies as described in Figure 1 and Methods. Full ANOVA statistics are in Table S5.

We tested food-deprived adults of the resulting 18 trans heterozygote genotypes and compared food-leaving scores of *for;mutant* to the *for;Balancer* which controlled for genetic background effects (see Methods). Recall that food-deprived homozygous rovers show low levels of food leaving behaviour (Figure 1) while sitters have higher levels. As expected, the control *for;Balancer* flies show the previously found lower level of behavioural response for rovers compared to sitters (Figure 6A and 6B, solid lines), indicating no direct effects of, or interactions with, the balancer chromosome background. There is, however, a significant epistatic interaction in the *for;InR* and *for;dp110* flies (Figure 6A and 6B dashed lines; Table S5). Rovers crossed to these insulin pathway mutants become more sitter-like. In contrast, the interaction with negative regulator *foxo* is not significant (Table S5). Mutants of positive regulators of insulin signaling make rover food-leaving behaviour more like sitters (reduces RNS), while a mutant of negative regulator *foxo* trended towards making rovers less like sitters (increases RNS). This suggests that there is a significant (epistatic) interaction between *for* and the two positive regulators of the insulin signaling pathway tested here. This is consistent with rovers experiencing greater shifts in insulin signaling effects between Fed and FD states than sitters. There are also differences between natural and mutant sitters in the interaction with *InR* (Table S5) suggesting that the difference in genetic backgrounds between these strains may also affect this interaction. For this behavioural measure, natural sitters are intermediate between rovers and mutant sitters, a difference from the trend found in gene expression overall (via RNS, Figure 5) or in regulators of insulin signaling (Figure S2). Thus the effect of the background difference between natural sitters and the other strains varies between gene expression and behavioural measures.

### Meta-analysis of insulin and *foraging* effects on gene expression

We performed a bioinformatic meta-analysis comparing our array results to those from three published microarray studies which manipulated insulin/Tor signaling [28],[29],[30]. This provides additional evidence for transcriptional parallels between *for* and insulin. We use these studies to identify sets of genes which were up- or down-regulated by the manipulation of insulin/Tor signaling, and which had high enough expression levels in our data for reliable comparison. To ensure independence of the three analyses we used sets of genes which did not overlap between studies (see Table S6 for gene selection criteria).

For each up- or down-regulated set of genes identified from a study we calculated the mean log_2_ fold change between rovers and mutant sitters when Fed or FD. This gave four comparisons per study (up/down regulated in study 1, 2 or 3×Fed/FD in our data). In other words, we used the 3 independent studies to tell us which genes may be transcriptionally regulated by insulin signaling. We then used our data to ask, for the same genes, what the rover-sitter difference in expression is under the two food conditions. Our hypothesis is that rovers have higher insulin signaling when Fed than sitters, but not necessarily when FD. Hence we predict that genes requiring insulin signaling for their expression should have higher expression levels in Fed rovers than in Fed sitters, but that this difference may not exist in FD rovers and sitters. Similarly, if genes are shown in the independent study to have lower expression when insulin signaling is high (or equivalently, higher expression when insulin expression is reduced), then we predict those genes should have lower expression in Fed rovers than in Fed sitters.

**Figure 7:**
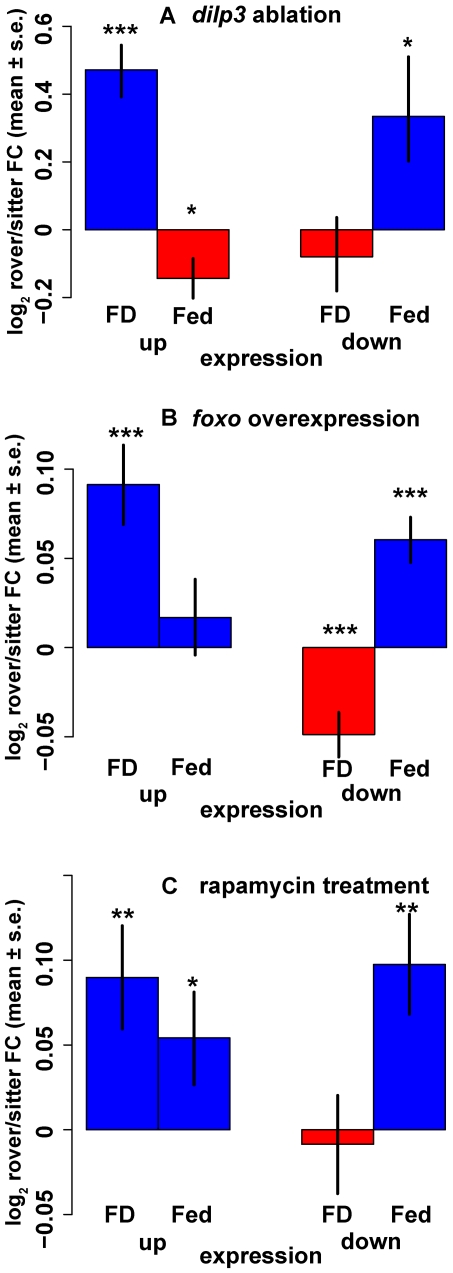
Meta-analysis of 3 manipulations of the insulin/Tor signaling identifies rover-biased genes. Three published studies decreased insulin/Tor effects via (A) ablation of *dilp3* expressing cells [28], (B) overexpression of constitutively active *foxo* [29], or (C) rapamycin [30]. We used data from these papers to identify sets of genes in each study whose expression went up or down in response to the particular insulin/Tor manipulation (Table S6 gives full statistics and methods). For each gene set (expression up or down), we plot average log_2_ fold change between rovers and mutant sitters in our study on the vertical axis, one bar for FD flies and one for Fed flies. When gene expression is reduced by insulin signaling (e.g. increases due to *dilp3*/*foxo*/rapamycin ablation), food deprived rovers have significantly higher mean expression than sitters (far left in each panel). When gene expression is increased by insulin signaling (e.g. decreases due to *dilp3*/*foxo*/rapamycin ablation), fed rover expression is higher than sitters (far right each panel). This was true for gene sets used from the ablation of *dilp3* expressing cells publication [28] (A), the overexpression of *foxo* publication [29] (B), and the rapamycin treatment paper [30] (C). This results in significant negative interactions (Table S6) for genes repressed by insulin signaling, and significant positive interactions for genes increased by insulin. Error bars = 1 s.e.m. Blue bars, mean rover expression is higher than sitter, red bars, mean sitter expression is higher than rover.

In the first study, Buch et al. [28] ablated *dilp3* secreting cells in adults and used microarrays to compare ablation lines which had reduced insulin signaling to that of controls. Figure 7A shows a summary of the four rover-sitter comparisons for this study. Bars on the right labelled “expression down” are for genes whose expression was reduced by *dilp3* ablation (i.e. insulin signaling increases expression of these) and bars on the left (“expression up”) are for genes whose expression was increased by *dilp3* ablation (genes repressed by insulin signaling). Genes with expression reduced by *dilp3* ablation (Figure 7A, left) show a negative GEI interaction sign *I* (rovers higher when food deprived, mutant sitters higher when fed). Conversely genes increased by *dilp3* ablation (Figure 7A, right) show a positive GEI interaction sign *I* (no difference when food deprived, rovers higher when fed), in accordance with our predictions.

Figure 7B gives the four comparisons for genes whose expression was changed by *foxo* overexpression [29]; Figure 7C is for genes changed by rapamycin treatment [30]. In each case the pattern is similar to *dilp3* ablation: genes with expression increased by a manipulation equivalent to lowering insulin/Tor signaling (genes reduced by insulin) show the negative *I* GEI interaction, while genes whose expression is reduced by the manipulation (genes increased by insulin) show positive *I*. Full statistics are given in Table S6. This table also shows that the pattern in *I* is more significant when natural sitters are used in the analysis than when only mutant sitters are used, so the trends shown in Figure 7 apply to both mutant and natural sitters.

In summary, the patterns of GEI interaction strength *I* in rover-sitter gene expression of genes affected by three different manipulations of insulin/Tor signaling in three independent studies [28],[29],[30] are consistent with our hypothesis in each study. Genes requiring insulin signaling for expression show positive rover-sitter *I* and genes inhibited by insulin signaling show negative rover-sitter *I*.

## Discussion

The *foraging* gene in *Drosophila* which encodes PKG is known for its importance as a natural variant affecting behavioural and neural plasticity [11],[14],[31],[32],[33],[34],[35]. We now demonstrate that it also affects metabolic, gene expression, and behavioural plasticity in adult flies. Specifically, rovers show a greater response to changes in their food environment than either mutant or natural sitters for the majority of behavioural, metabolite, and gene expression traits studied here. The pattern of such changes is matched by the pattern of expression of positive regulators of insulin signaling. Combining *for* alleles with mutants of positive insulin signaling regulators makes rover responses sitter-like, but does not change sitter responses. Collectively these findings suggest that the effect on metabolic, gene expression, and behavioural plasticity of *foraging* works in part through the insulin signaling pathway.

The *for* product PKG and the insulin pathway are conserved across many animals, from worms to flies and mammals [36]. PKG has been found to produce behavioural responses to food in flies [14],[33], nematodes [37],[38], honeybees [39], and ants [40],[41]. In particular, PKG interacts with insulin and TGF-beta signaling in worms to regulate quiescence, a state possibly related to satiety [37]. In this study we have focused on the insulin pathway, but potential interactions between *for* and TGF-beta may be a fruitful area for future study in flies.

### GEI, *foraging*, insulin, and energy store allocation

Expression of insulin pathway genes such as *InR* and *Pi3k92E (Dp110)* is inversely related to the strength of insulin signaling via the *foxo* transcription factor: *foxo* is retained in the cytoplasm when signaling is high, but translocates to the nucleus and stimulates transcription of pathway genes when signaling is low [23],[24]. Since insulin gene expression is opposite to insulin signaling strength, our finding of greater negative transcriptional effects on insulin pathway genes in rovers (Figure S2) suggests the presence of greater positive insulin signaling in rovers. Since insulin signaling upregulates anabolism and reduces catabolism [19],[42], this is consistent with the patterns we find in genes involved in anabolic (fed rover-biased) and catabolic (fed sitter-biased) processes. And genes which require insulin for expression are higher in fed rovers, while genes repressed by insulin tend to be higher in fed sitters (Figure 7).

The finding of genetic interactions between *for* and genes in the insulin signaling pathway raises questions for future investigation. Insulin signaling in flies can reduce flow through tricarboxylic acid cycle and oxidative phosphorylation and increase flow through the pentose-phosphate shunt, freeing pyruvate and acetyl CoA for lipogenesis and increasing NADPH and precursors for biosynthesis [43]. This is consistent with patterns in the Fed adult rover catabolic groups (Figure 4C, Table S3). Instead of accumulating energy as fat, Fed adult sitters accumulate carbohydrates. Because of the lower density of fat and its higher caloric content, rovers store more energy per unit mass than sitters, a difference which should have implications for life history characteristics such as starvation resistance (see below).

Several studies note changes in fat stores in flies with mutations in insulin signaling genes. These include, loss of fat in *melted* mutants [44], gain of fat in *InR*, *chico* and Pi3K (also called *Dp110*) mutants [45],[46] and Pi3K-overexpression in larvae increases accumulation of nutrients in fat [47]. Nuclear *foxo* reduces fat, phenocopying starvation [44],[48], and it reduces head fat body insulin signaling [49]. Thus, there may be multiple different effects of insulin-related genes on fat. Could *foxo* mediate lower sitter fat levels? Rapamycin treatment (which acts downstream of *foxo* specifically on the Tor signaling pathway) also produces similar patterns of effects (Figure 7C). Hence, indirect effects of insulin signaling on the Tor pathway could also be involved. In support of this, *PDK/Pk61C* is the gene in the insulin pathway showing strongest transcriptional regulation in rovers versus both natural and mutant sitters (Figure S2B). PDK phosphorylates ribosomal S6 kinase (*S6k*), part of Tor regulation of translation [20],[21],[50]. Indeed, overexpression of Tor has been shown to increase triglycerides in adult male flies [44].

Genes repressed by *foxo*, rapamycin, or *dilp3* ablation are rover-biased in Fed flies, while genes increased by insulin/Tor knockdown are rover-biased in FD flies (Figure 7A–7C); that is, if a gene's expression is increased by insulin/Tor signaling, it tends to be higher in Fed rovers, while if it's expression is increased by inhibition of insulin/Tor signaling, it tends to be higher in FD rovers. This is an example of the more general trend illustrated in Figure 5, where the change between Fed and FD flies is larger in rovers than in sitters for behaviours (89%), metabolites (84%), genes (77%), and gene groups (77%). In mammals, a reduced physiological response to food is a sign of insulin resistance; whether this is true of sitters waits further testing.

Our data provides direct evidence through genetic crosses and considerable correlational information through patterns of insulin gene expression and meta-analysis that the GEI effects of *foraging* in Fed and FD flies are mediated at least in part through interaction with the insulin/Tor signaling pathways.

### Consequences of lipid–carbohydrate allocation differences

Whole-body energy stores in fed rovers and sitters differ: how might fatty rovers and starchy sitters differ in life-history? A number of life-history and ecological parameters have been shown to be related to lipid or carbohydrate reserves in flies, including flight capacity, starvation and desiccation resistance. Diptera in general and fruit flies in particular are dependent on glycogen reserves and hemolymph sugars to fuel flight muscles [51],[52],[53]. Glycogen phosphorylase, which has strong GEI in rovers and sitters, is a rate limiting enzyme for glycogen mobilization to support flight [54] (Figure 4A). Flies selected for postponed ageing show increased flight duration, glycogen reserves and resistance to desiccation, [55], while desiccation-selected flies show higher glycogen levels [56]. Glycogen, desiccation resistance, longevity and stress resistance may form a cluster of correlated traits in flies [57]. Lipid content of adult flies correlates with starvation resistance [58]; among lifespan-selected and other lines, starvation resistance was correlated with lipid content and not glycogen [55]. This correlation extends to sibling species *D. simulans* [59]. In a cricket species where lipids can be used to support flight, a trade-off between lipid reserves for flight and for egg production has been reported [60],[61].

The rover-sitter system, with its dichotomous Y-allocation [62] of energy stores to lipids and carbohydrates, may therefore be useful for studying single-gene influences on traits with costs and benefits associated with energy use and storage including flight capacity, desiccation and starvation resistance.

### GEI due to *foraging* and neuromuscular function

The patterns of GEI and *I* between *foraging* and food (Fed vs. FD) are very consistent for genes whose primary function is in anabolic or catabolic pathways, with *I* positive in anabolic groups and negative in catabolic groups. However, there are many more genes with significant *for* GEI. An important set of such genes is involved in nerve and/or muscle function (Table S3). PKG affects synaptic plasticity in mammals [63],[64],[65],[66] and learning and memory in flies [33],[34].

The possibility that PKG may cause GEI through its role in regulating ion homeostasis in nerves and muscles deserves further examination. PRKG1, the mammalian homolog of *for*, regulates calcium and potassium fluxes in smooth muscle relaxation where it is associated with the myosin phosphatase complex, Ca^++^ATPases, and potassium ion channels [67]. We find a cluster of gene groups with *I*<0 associated with muscle and actin cytoskeleton, including genes such as *wupA* (troponin-I) and *Prm* (paramyosin). These are some of the genes whose expression is most correlated with *for* in a coexpression analysis across humans, flies, worms, and yeast [68].

Calcium/potassium levels are important in synaptic function and plasticity, and mutations in potassium channel genes affect habituation in the giant-fiber axon escape reflex in flies [69],[70]. Habituation of the giant-fiber escape reflex differs in adult rovers and sitters [31]. Rover-sitter differences in PKG are also associated with different voltage-dependent K^+^ currents in larval neuromuscular junctions, along with differences in neuronal excitability, neurotransmitter release, and synaptic transmission [71]. Rover-sitter differences in neural thermotolerance arise from differences in the regulation of K^+^ channel activity via a circuit involving PKG, PP2A, and ion channels [72]. Thus our demonstration of rover-sitter differences in gene expression of genes involved in neurotransmitter release, postsynaptic membranes, and calcium- and potassium-channels supports previous studies. It will be important to determine whether *foraging* interacts epistatically with other genes influencing K^+^ currents in neurons and muscles. It is also of interest to investigate whether the metabolic effects of allelic variation in *for* are independent of, or are tied to PKG's effects on ion homeostasis and neural function.

### Magnitude of *foraging* GEI compared to effects of other genes

Our study used only a few strains of flies and thus does not speak to the importance of *for*-mediated effects on the genome in natural populations. However, we are able to consider allelic effects at the *for* locus relative to the genetic background effects in a principal components analysis which identifies genetic background (BG) and food (Fed vs. FD) as the most important factors, followed by the interaction of BG and food, *for* genotype main effects, and *for* interaction effects. Using the Storey-Tibshirani method to estimate the true proportion of differentially expressed genes π_alt_ shows that *for* GEI affects 57% of the highest expression genes. We also found that the effect of the natural sitter background was to intensify gene expression contrasts with rovers but to reduce behavioural contrasts. Thus, an important future step is to quantify the relative importance and roles of *for* and other genes in a wider variety of natural genetic backgrounds.

Our results also speak to evolutionary questions about pleiotropy, epistasis, and plasticity. Pleiotropic genes may affect few traits when redundancy, degeneracy [73], or compensations in gene networks buffer the effects of mutations [74], while mutations in other genes produce large changes [75]. The number of traits influenced by a gene follows a power law, with a few genes having widespread affects [74],[76]. It has been proposed that the use of naturally occurring alleles or mild mutations is more relevant to studies of epistasis and network stability than the more common use of knockouts or severe loss of function mutations [73],[77],[78]. The question of whether some genes can increase phenotypic plasticity and thus whether selection can act to increase or decrease plasticity has been the subject of much debate [2]. In *for* we have an example of a gene with naturally occurring alleles maintained in a stable polymorphism in the wild [79]. We demonstrate that in adult flies *for* interacts pervasively with food, producing pleiotropic GEI in behaviours, lipids and carbohydrates, and gene expression. Our quantitative plasticity measure reveals that *for*×food GEI is often due to rovers having significantly higher food-related plasticity than sitters. We show that *for* interacts with genes of the insulin signaling pathway to produce some of these effects. The *foraging* gene may thus provide a suitable context for resolving some of these questions relating to phenotypic plasticity and selection.

## Methods

### Strains and rearing

The following allelic variants of the chromosome-2 *foraging* (*for*) gene were used in this study: the *for^R^* (natural rover), *for^s^* (natural sitter) variants and *for^s2^* (a sitter mutant strain made on a *for^R^* genetic background) [9],[13]. All strains share common isogenic third chromosomes from *for^R^* and common X chromosomes. Genetic variation on the small fourth and Y chromosomes was not controlled. For tests of epistasis we used three null mutants of genes in the insulin signalling pathway (see below).

Flies were reared on medium b for behavioural assays, gene array and metabolite experiments (Table S1A) [80]. For additional behavioural assays reported in Table S1B, S1C flies were raised in media described in Table S1A, S1C. Flies were raised in 40 mL plastic vials containing 10 mL food medium in 12/12 h light/dark cycle (lights on 08:00), 25±1°C, 70±5% relative humidity (standard conditions).

### Fly rearing and behaviour testing

Flies were collected 0–2 days post eclosion, separated under light CO_2_ anaesthesia, then reared in groups of 25–30 for 4 days. 12–13 males and 12–13 females were used in each rearing group. Adult rearing was done under standard conditions as described above. Flies were transferred to test media the night before behaviour tests. Test media consisted of 10 mL of food medium (Fed), or 10 mL 1% agar for food deprivation (FD) tests in vials. Flies were tested in the morning (9–12 a.m.) after 16–18 hours under Fed or FD conditions.

A plexiglass maze was used for the food-leaving assay; the maze is as described [80] and is shown in Figure S1. Each morning mazes were conditioned by passing through one sample of 25 natural sitter flies before testing commenced. Mazes were placed horizontally on a light table with a uniform light intensity of 1000 lumens. Flies were placed in a10×75 mm borosilicate glass tube (the “sugar entry tube”) containing 0.5 mL 0.25 M sucrose in 1% agar for 15 min prior to test. At start of testing, the sugar tube with flies is placed in the entry port of the maze. Empty glass collection tubes are placed in the 9 exit ports of the maze. After 3 min, flies in collection tubes at exit ports are counted, as are flies remaining in the sugar tube. Food-leaving score is (flies in collection tubes)/(total flies). All treatment conditions were tested on at least 3 different test days.

### Quantitative complementation crosses

To test whether the rover and sitter *for* alleles interact epistatically with null alleles of the three genes involved in the insulin signaling pathway we used a form of quantitative complementation, a method of complementation developed for testing quantitative effects, in this case, gene interactions [27].We asked if one copy of a mutant allele of a gene involved in insulin signaling, in the presence of each of the *for* alleles, changes food-leaving behaviour. Crosses were made between rover (*for^R^*), natural sitter (*for^s^*), and mutant sitter (*for^s2^*) strains and balanced loss of function mutants in three insulin signaling pathway genes: (a) *InR*, the insulin receptor (mutant allele: *InR^93dj^* ^-4^ [81]); (b) *Pi3K92E/Dp110*, the phosphatidylinositol 3-kinase catalytic subunit (*Dp110^B^* [25]); and (c) *foxo*, (*foxo^21^* [22]). Strains carrying mutations in the insulin signaling pathway were: (a) *In(3R)GC25*, *InR^93Dj-4^/TM3*, *Sb^1^* (Bloomington stock 9554) [82]; (b) *yw*;*P[ry* ^+^,*gH]*, *Dp110^B^*/*TM3*,*Ser*,*y* ^+^(*C*) [83]; (c) *foxo^21^/TM6C* [22]. All mutants of the insulin signaling pathway were maintained heterozygous with balancer chromosomes which did not carry mutations in these insulin signaling genes and were on a sitter background. Epistasis is identified as a two-way statistical interaction between the variant (rover or sitter) and test genotype (mutant or control) [26] (see below). The balancer heterozygotes control for effects of natural variation in the genetic background that can result in increases or decreases in food leaving behaviour.

### Metabolite analysis

Fourier Transform Ion Cyclotron Resonance Mass Spectrometry (FTICR MS) was used to analyze homogenized fly heads (equal numbers of males and females) from 5–7 day post eclosion *for^R^* and *for^s2^* strains harvested in the morning. Food Deprived (FD) flies had been restricted to water in agar 12 hours before collection. Samples were taken in triplicate. Values shown are Signal to Noise (S/N) ratios. Each sample was analysed as described [84] using the DiscovaMetrics [85] package producing parent ion molecular weights accurate to within 0.0005 daltons; compound identifications were cross-checked against Kegg Ligand [86] and Metlin [87] databases. FTICR MS has maximum sensitivity to metabolites in the 100–1000 Dalton range, accuracy of 0.0001 Dalton, and uses six different buffer/ionization modes (Table S2) each detecting different classes of metabolites. For instance, compounds such as sugars and phosphates are detected in mode 1102 using a polar buffer and negative ion electrospray, while mode 1203 uses a non-polar buffer and positive atmospheric pressure chemical ionization mode to detect compounds such as triacylglycerols.

Whole body lipid and carbohydrate analysis was performed separately on males and females of 5–7 day post-eclosion *for^R^*, *for^s^*, and *for^s2^* strains. For both lipid and carbohydrate analyses, results were standardized against dry weight. For lipids, the ether extraction method was used as described [88]. In brief, flies were frozen in liquid nitrogen, then weighed to the nearest 0.01 mg in groups of 5–10 flies on a Mettler Toledo XS205 balance. Flies were then dried at 60°C for 24 hours and reweighed. Lipids were then extracted in 1 mL of ether for 24 hours, after which ether was decanted and flies were dried at 60°C for 24 hours and weighed. Total carbohydrate levels were determined using amyloglucosidase digestion followed by spectrophotometric determination of total glucose using NAD to NADH reduction [89]. Sigma kit GAHK20. Briefly, hexokinase catalyzes phosphorylation of glucose in the presence of ATP to Glucose-6-phosphate (G6P), which is then oxidized to 6-phospho-gluconate in the presence of oxidized nicotinamide adenine dinucleotide (NAD) in a reaction catalyzed by glucose-6-phosphate dehydrogenase (G6PDH). During this oxidation, an equimolar amount of NAD is reduced to NADH. The consequent increase in absorbance at 340 nm is directly proportional to glucose concentration. Protein was measured using the bicinchonic acid (BCA) method. Total energy content was calculated based on ratios of 9∶4∶4 Kcal/gm for fats∶carbs∶protein.

### Microarray analysis

Affymetrix Drosophila Genome 1.0 cDNA microarrays were used to evaluate effect of *foraging* genotype and feeding state on transcript levels in adult heads. Flies homozygous for each of the 3 alleles, *for^R^*, *for^s^*, and *for^s2^*, were raised to 5–7 days post-eclosion, and given Food or FD treatments as described above. Samples of flies (equal numbers of males and females) were frozen in liquid nitrogen, and heads were separated by sieving. RNA was extracted as described [90]. Triplicate RNA samples for each treatment were hybridized to Drosophila Genome 1.0 microarrays, for a total of 18 arrays. N = 3 within each treatment. Expression levels produced by MAS 5.0 were normalized by quantile normalization [91] of log_2_ transformed data. Full *MIAME* information and expression set data is filed as GEO accession GSE14371. Pathway analysis and gene ANOVA were performed as described in Statistical methods. Analysis of variance was used to detect significant GEI for individual genes after False Discovery Rate correction for multiple testing [17].

### qRTPCR analysis

Levels of expression of 2 genes (*Treh*, *CG10924*) were confirmed by quantitative reverse transcriptase polymerase chain reaction analysis (Figure S3, Text S1). Heads of male and female *for^R^* and *for^s2^* flies were raised as in the microarray analysis and then were frozen in liquid nitrogen. RNA was extracted using the Trizol method (15596-018, Life Technologies) and further purified using the Qiagen RNeasy kit (74106, Qiagen). The amount of RNA in each sample was determined using a Nanodrop spectrophotometer (ND-1000) and sample quality verified using 260/280 micron absorbance ratios.

### Statistical methods

Scores of each trait are analysed with two-way Analysis of Variance (ANOVA) to determine whether significant GEI exists (see detailed procedures below). The Storey-Tibshirani False Discovery Rate (FDR) [17] is used for multiple testing correction and estimation of π_0_ using the *qvalue* package as implemented by Storey [17], with default parameters. Thus ANOVA *p* values have been replaced by FDR *q* values, and *q*<0.05 is deemed significant.

RNS measures which strain has higher plasticity and is defined as RNS = (|rover change|−|sitter change|)/C (for log_2_ transformed data C = 1, else C = mean of all treatments). That is, RNS compares the absolute magnitude of changes in response to food and is positive when rovers change more than sitters.

For ANOVA of gene expression data with two sitter strains and one rover, a modified general linear model design matrix was used (Text S1, Supplementary Methods) to ensure unbiased estimation. Briefly, factors RS (rover or sitter), food (Fed or FD), and BG (genetic background, 2 levels, 1 for rovers and mutant sitters, another for natural sitters) were analysed including main effects and the interactions RS×food and BG×food. A reduced model omitting the interaction BG×food was also fitted. For each gene, the first and second models were compared using Schwartz's Bayesian Information Criterion (BIC) [92] to determine whether to report full or reduced model results. Thus if interaction of BG and food was significant (as determined by BIC) we reported statistics from the full model, else from the reduced model. FDR correction was then applied to p-values from the selected model.

For group-wise ANOVA analysis of groups of metabolites or genes, a linear model as above, with the addition of a factor G with one level for each gene or compound was used; this is similar to adjusting each gene or compound to have a mean of zero, but accounts more conservatively for lost degrees of freedom due to the adjustment. Only transformed variables with approximately equal variances are used in group-wise ANOVAs. Microarray data is log_2_ transformed, then subjected to quantile normalization and a variance-equalizing monotonic transform. After these steps variances for the top-expressing 60% of genes were approximately equal and data was normally distributed. For group-wise ANOVA of metabolites, log_2_ data was used. Data was tested with a covariate of molecular weight (MW). If MW or its interactions with food and genotype were significant, the ANCOVA with MW is reported; otherwise group-wise ANOVA results are reported.

For ANOVA of behavioural experiments where behaviours may vary from day to day (Day effect), Day was added as a random factor to the ANOVA described above for single genes, and significance of this mixed-model was determined by F-tests of fixed factor terms to their interactions with Day. See Table S1 and Table S5 for examples.

In the quantitative complementation crosses in Table S5, the interaction of *for* with a factor representing the presence or absence of the mutant insulin gene is tested. That is, we test for epistasis rather than GEI. The ANOVA analysis is identical in format to that just described, with the factor representing presence/absence of insulin mutant replacing the food factor.

## Supporting Information

Figure S1 Behaviour testing apparatus. A plastic maze originally used for geotaxis experiments [80] is placed horizontal on a light table adjusted to produce 1,000 lumens illumination. Darker areas on photograph edges are due to camera contrast adjustment; actual illumination is even over maze surface. The entry tube contains agar with 0.25 M sucrose (Methods). 24–26 flies are placed in this tube 15 minutes before entry to the maze. 9 empty (no agar or sugar) collection tubes block exit points from maze. At time 0 the entry tube is placed in the maze entry. Numbers of flies in collection tubes is counted every minute until termination of run at 3 minutes. Experiments were conducted in a darkened room maintained at 25 C and humidified to 60%RH.
(2.47 MB TIF)

Figure S2 Positive regulators of insulin signaling - rovers change expression more than sitters. (A) Group mean expression. The average log_2_ expression of a group of positive regulators of insulin signaling (*InR*, *chico/dIRS*, *Pi3K92E/dPIK3CB/dp110*, *Pi3K21B/dp60*, *Pk61C/dPDK*, *Akt1*) is shown for rovers (blue), mutant sitters (red), and natural sitters (pink) in two food environments. Average expression shows strong negative *I* or GxE interaction - that is, rovers show the downregulation expected [23],[24] in Fed flies much more than sitters (*for×Food F_1,97_ = 15.52*, *p* = 0.00015, group ANOVA). Natural sitters have a different genetic background (BG) from rovers and mutant sitters. The effect of the BG is to strengthen the negative GEI (*I* = −0.33, rover vs sitter mutant; *I* = −0.48, R vs s; *BG×food F_1,97_ = 9.42*, *p* = 0.0029). RNS (plasticity of response) is positive - rovers change more than sitters. Error bars are ±1 s.e.m. (B) Range of Individual gene expression between Fed and food deprived (FD) heads. Expression of positive regulators of insulin signaling tends to be higher in the food-deprived state due to *foxo*-mediated upregulation of transcription [23],[24]. The vertical axis shows log_2_ fold change between Fed and FD flies.
(1.36 MB TIF)

Figure S3 qRTPCR results. qRTPCR was done with extracts from heads of *for^R^* (blue) and *for^s2^* (red) flies using actin (*Act57B*) as a reference gene for triplicate samples from fed and food deprived (FD) flies. Data are normalized to fed *for^s2^* levels and shown as log_2_ values. (A,B) Trehalase (*Treh*) PCR and array; (C,D) phosphoenolpyruvate carboxykinase (GTP) (*CG10924*). Pearson's correlation between array and qPCR values: *Treh t* = 105.9, df = 1, p = 0.006; *CG10924 t* = 31.8, df = 1, p-value = 0.02. See Text S1 (Supplementary Methods) for details of qRTPCR extraction.
(1.80 MB TIF)

Table S1 Analysis of variance of behaviour and four food media.
(0.12 MB DOC)

Table S2 FTICR MS metabolite data.
(0.07 MB DOC)

Table S3 Gene groups with significant GEI.
(0.23 MB DOC)

Table S4 Relative Nutrient Sensitivity (RNS) for metabolites.
(0.06 MB DOC)

Table S5 Complementation cross analysis of variance for insulin mutants.
(0.09 MB DOC)

Table S6 Meta-analysis: genes that respond to insulin differ between rovers and sitters.
(0.14 MB DOC)

Text S1 Supplementary methods.
(0.05 MB DOC)
