# Supplementary figures and images for: The Drosophila foraging Gene Mediates Adult Plasticity and Gene–Environment Interactions in Behaviour, Metabolites, and Gene Expression in Response to Food Deprivation
Source: PLoS Genet. 2009 Aug 21;5(8):e1000609. doi: 10.1371/journal.pgen.1000609 (PMC2720453; doi:10.1371/journal.pgen.1000609)

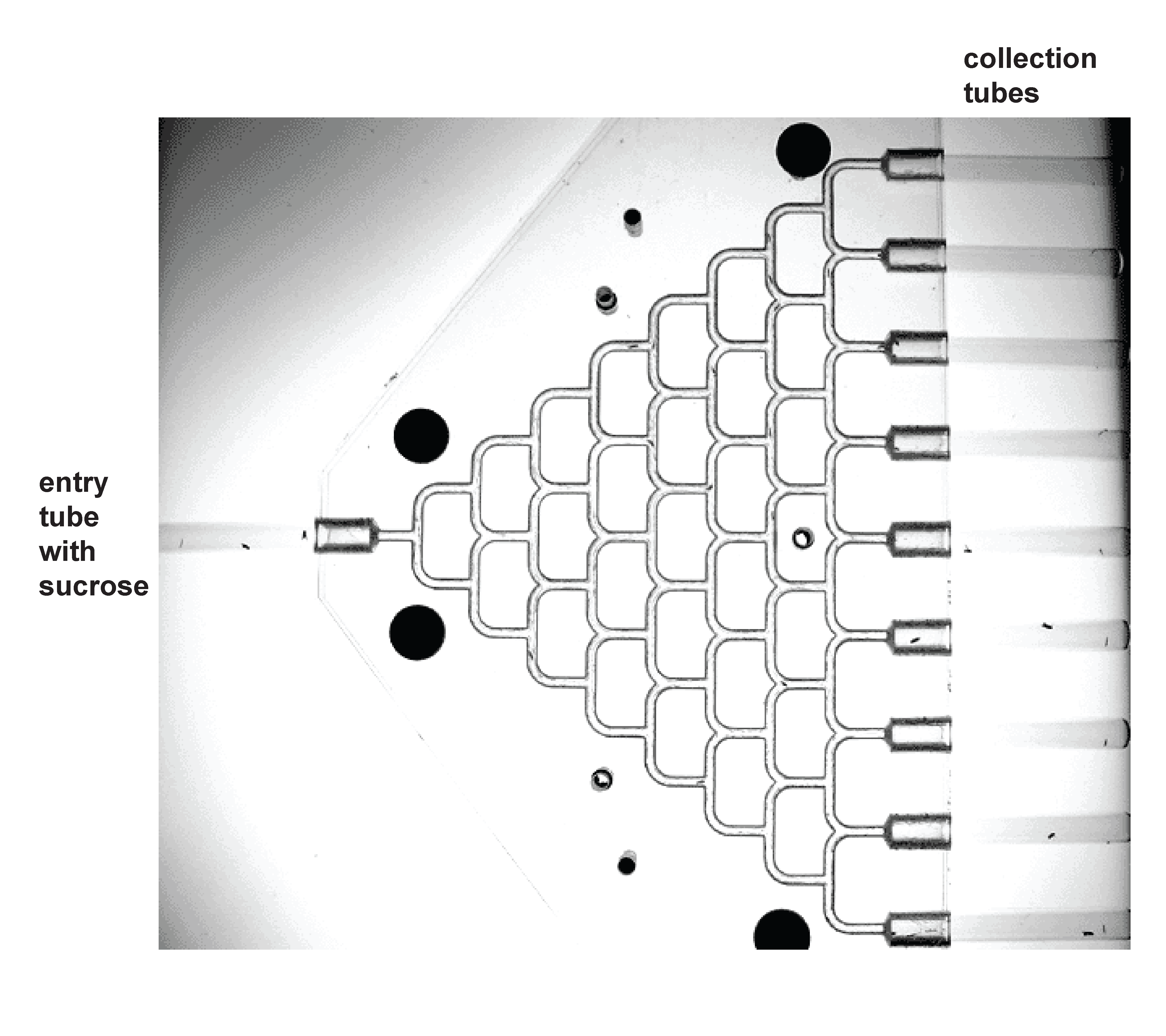

Supplement: Figure S1 — Behaviour testing apparatus. A plastic maze originally used for geotaxis experiments [80] is placed horizontal on a light table adjusted to produce 1,000 lumens illumination. Darker areas on photograph edges are due to camera contrast adjustment; actual illumination is even over maze surface. The entry tube contains agar with 0.25 M sucrose (Methods). 24–26 flies are placed in this tube 15 minutes before entry to the maze. 9 empty (no agar or sugar) collection tubes block exit points from maze. At time 0 the entry tube is placed in the maze entry. Numbers of flies in collection tubes is counted every minute until termination of run at 3 minutes. Experiments were conducted in a darkened room maintained at 25 C and humidified to 60%RH. (2.47 MB TIF) [file pgen.1000609.s001.tif]

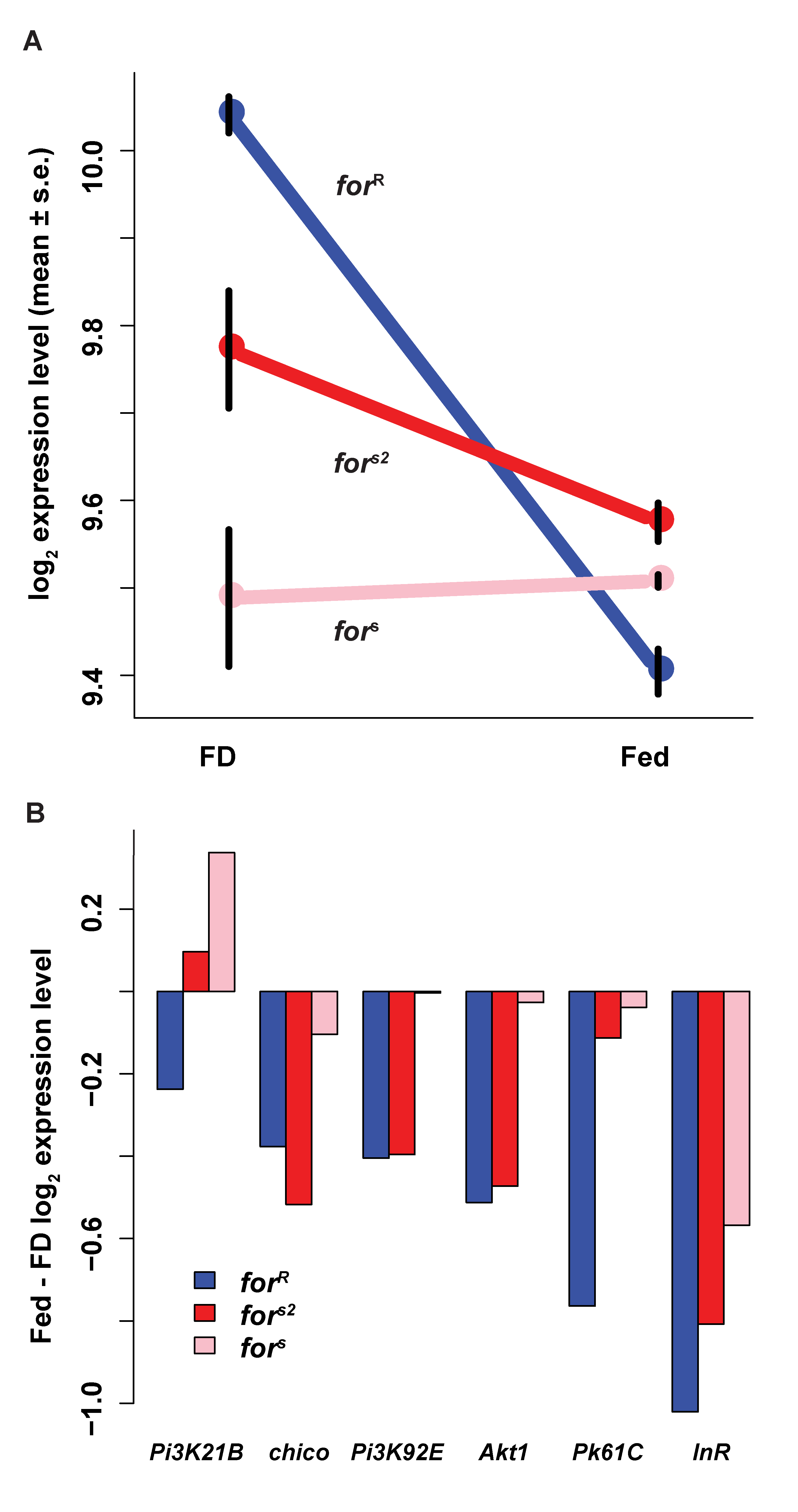

Supplement: Figure S2 — Positive regulators of insulin signaling - rovers change expression more than sitters. (A) Group mean expression. The average log2 expression of a group of positive regulators of insulin signaling (InR, chico/dIRS, Pi3K92E/dPIK3CB/dp110, Pi3K21B/dp60, Pk61C/dPDK, Akt1) is shown for rovers (blue), mutant sitters (red), and natural sitters (pink) in two food environments. Average expression shows strong negative I or GxE interaction - that is, rovers show the downregulation expected [23],[24] in Fed flies much more than sitters (for×Food F1,97 = 15.52, p = 0.00015, group ANOVA). Natural sitters have a different genetic background (BG) from rovers and mutant sitters. The effect of the BG is to strengthen the negative GEI (I = −0.33, rover vs sitter mutant; I = −0.48, R vs s; BG×food F1,97 = 9.42, p = 0.0029). RNS (plasticity of response) is positive - rovers change more than sitters. Error bars are ±1 s.e.m. (B) Range of Individual gene expression between Fed and food deprived (FD) heads. Expression of positive regulators of insulin signaling tends to be higher in the food-deprived state due to foxo-mediated upregulation of transcription [23],[24]. The vertical axis shows log2 fold change between Fed and FD flies. (1.36 MB TIF) [file pgen.1000609.s002.tif]

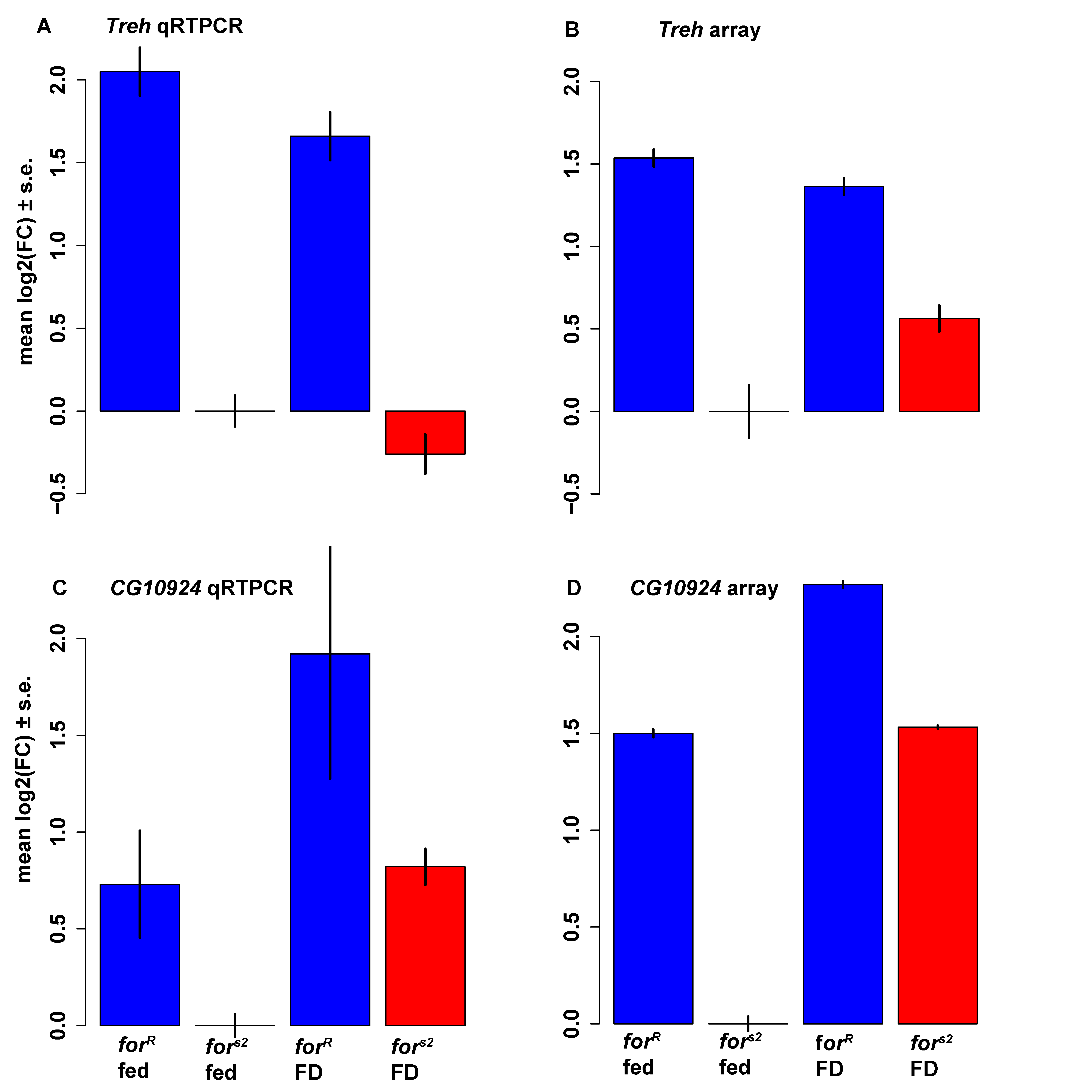

Supplement: Figure S3 — qRTPCR results. qRTPCR was done with extracts from heads of forR (blue) and fors2 (red) flies using actin (Act57B) as a reference gene for triplicate samples from fed and food deprived (FD) flies. Data are normalized to fed fors2 levels and shown as log2 values. (A,B) Trehalase (Treh) PCR and array; (C,D) phosphoenolpyruvate carboxykinase (GTP) (CG10924). Pearson's correlation between array and qPCR values: Treh t = 105.9, df = 1, p = 0.006; CG10924 t = 31.8, df = 1, p-value = 0.02. See Text S1 (Supplementary Methods) for details of qRTPCR extraction. (1.80 MB TIF) [file pgen.1000609.s003.tif]
