# Supplementary material for: The Drosophila foraging Gene Mediates Adult Plasticity and Gene–Environment Interactions in Behaviour, Metabolites, and Gene Expression in Response to Food Deprivation
Source: PLoS Genet. 2009 Aug 21;5(8):e1000609. doi: 10.1371/journal.pgen.1000609 (PMC2720453; doi:10.1371/journal.pgen.1000609)
Supplement: Table S1 — Analysis of variance of behaviour and four food media. (0.12 MB DOC) [file pgen.1000609.s004.doc]

# Supplementary Table 1. Analysis of variance of behaviour and four food media

**(a) Food medium compositions**

A standard water, agar, and minerals base was used in all foods (1,000 ml H2O, 10 g agar, 8 g C4H4KNaO6, 1g KH2PO4, 0.5g NaCl,0.5 g MgCl2, 0.5g CaCl2, 0.5g Fe2(SO4)3). The manufacturer’s reported analysis of yeast per 100 g dry yeast was 50 g protein, 5.3 g fat, 33 g carbohydrate. Media b,c,e contained a commercial dark corn syrup for which the manufacturer reported 125 g total carbohydrates/ 100 ml from an unspecified mixture of glucose, fructose, and blackstrap molasses. Medium b was used for flies in all main figures, including behaviour, metabolite, gene array, and quantitative complementation cross tests. Other media were used to investigate sensitivity of behaviour to food (see below).

| Medium | sucrose/L | syrup/L | yeast/L |
| --- | --- | --- | --- |
| a | 50 g | 0 | 25 g |
| **b** | **15 g** | **30 ml** | **35 g** |
| c | 87.5 g | 30 ml | 35 g |
| d | 100 g | 0 | 50 g |
| e | 116.3 g | 15 ml | 17.5 g |
| f | 50g | 0 | 0 |

**(b) Mixed model ANOVA for food leaving behaviours.**

See table for food (b) below for analysis of Figure 1 behaviour data. Data for behaviour tests on 3 other media (a,c,d) are also given here. Data for each food was first tested with a full mixed model ANOVA including terms for genetic background differences and their interaction with food, as an approximate test of whether the genetic background difference between the strains contributes to GEI (See Statistical Methods). Day of testing was the random factor, genotype and food presence/absence were fixed factors. For none of the four foods was there significant GEI between background and food (p>0.5 for all four tests; data not shown). Hence (see Statistical Methods) the results of a reduced mixed ANOVA model with fixed factors rover/sitter type (labeled *for*), food presence/absence (label: food), genetic background (label: BG) and random factor Date of testing are presented (see Statistical Methods for detailed model). Significance of fixed terms is tested against their interaction with the random factor Date.

| Food (a) | *df* | *MS* | *df x Date* | *MS x Date* | *F* | *p* |
| --- | --- | --- | --- | --- | --- | --- |
| *for* | 1 | 0.179 | 4 | 0.0042 | 42.37 | **0.0029** |
| *food* | 1 | 0.377 | 4 | 0.0276 | 13.64 | **0.021** |
| *BG* | 1 | 0.024 | 4 | 0.0079 | 3.03 | 0.157 |
| ***for x food*** | 1 | 0.168 | 4 | 0.0131 | 12.82 | **0.023** |
| *Date* | 4 | 0.024 |  |  | 1.62 | 0.203 |
| *Residual* | 22 | 0.0148 |  |  |  |  |

**Food medium b is used for main figures, arrays, metabolites, and crosses.**

| Food (b) | *df* | *MS* | *df x Date* | *MS x Date* | *F* | *p* |
| --- | --- | --- | --- | --- | --- | --- |
| *for* | 1 | 0.019 | 6 | 0.0028 | 6.63 | **0.042** |
| *food* | 1 | 0.258 | 6 | 0.0081 | 31.69 | **0.0013** |
| *BG* | 1 | 0.000 | 6 | 0.0074 | 0.002 | 0.968 |
| ***for x food*** | 1 | 0.060 | 6 | 0.0063 | 9.65 | **0.021** |
| *Date* | 6 | 0.020 |  |  | 2.44 | 0.060 |
| *Residual* | 21 | 0.0080 |  |  |  |  |

| Food (c) | *df* | *MS* | *df x Date* | *MS x Date* | *F* | *p* |
| --- | --- | --- | --- | --- | --- | --- |
| *for* | 1 | 0.001 | 5 | 0.0042 | 0.16 | 0.709 |
| *food* | 1 | 0.102 | 5 | 0.0276 | 12.99 | **0.023** |
| *BG* | 1 | 0.027 | 5 | 0.0079 | 5.43 | 0.145 |
| ***for x food*** | 1 | 0.134 | 5 | 0.0131 | 18.80 | **0.023** |
| *Date* | 5 | 0.039 |  |  | 7.81 | **0.003** |
| *Residual* | 10 | 0.0050 |  |  |  |  |

| Food (d) | *df* | *MS* | *df x Date* | *MS x Date* | *F* | *p* |
| --- | --- | --- | --- | --- | --- | --- |
| *for* | 1 | 0.207 | 4 | 0.0172 | 12.06 | **0.025** |
| *food* | 1 | 0.012 | 4 | 0.0154 | 0.78 | 0.427 |
| *BG* | 1 | 0.001 | 4 | 0.0058 | 0.25 | 0.647 |
| ***for x food*** | 1 | 0.092 | 4 | 0.0100 | 9.14 | **0.039** |
| *Date* | 4 | 0.011 |  |  | 1.03 | 0.409 |
| *Residual* | 22 | 0.0109 |  |  |  |  |

**(c) Measured *I* values in 9 nutritional environments**

We performed behaviour tests on flies raised in 9 different nutritional environments (NE). NE is defined as a specific combination of larval food medium and adult food medium (food media in 1a, above; NE combinations below).

We held the effects of larval nutritional environment constant by raising animals from egg to eclosion on medium (d), and then from eclosion to time of testing on one of medium a-d to produce NE 1-4, below. Behaviour ANOVA for these NE are given above (1b)

Additional experiments for NE 5-9 were performed with smaller numbers of replicates so we do not report statistical analyses for these. However we did calculate the value *I* from arc-sine transformed scores for each environment for rovers versus mutant sitters (R-s2) and rovers versus natural sitters (R-s). The null hypothesis that *I*=0 over all 9 nutritional environments is rejected by a signs test, since all *I* values were positive.

| Nutr. env. | Larval food | Adult food | *I* (R-s2) | *I* (R-s) |
| --- | --- | --- | --- | --- |
| 1 | d | a | 0.155 | 0.138 |
| 2 | d | b | 0.288 | 0.199 |
| 3 | D | c | 0.298 | 0.205 |
| 4 | D | d | 0.171 | 0.210 |
| 5 | D | e | 0.009 | 0.274 |
| 6 | D | d,f† | 0.242 | 0.181 |
| 7 | A | a | 0.268 | 0.197 |
| 8 | B | b | 0.058 | 0.030 |
| 9 | C | c | 0.029 | 0.029 |

† NE 6 maintained flies on medium d until the night before testing. Flies in the FD treatment had access to water in agar only as usual while Fed flies had access to medium f (sucrose agar).
