# Supplementary material for: The Drosophila foraging Gene Mediates Adult Plasticity and Gene–Environment Interactions in Behaviour, Metabolites, and Gene Expression in Response to Food Deprivation
Source: PLoS Genet. 2009 Aug 21;5(8):e1000609. doi: 10.1371/journal.pgen.1000609 (PMC2720453; doi:10.1371/journal.pgen.1000609)
Supplement: Table S2 — FTICR MS metabolite data. (0.07 MB DOC) [file pgen.1000609.s005.doc]

# Supplementary Table 2. FTICR MS metabolite data

**(a)** Total compound levels per group **(b)** Group-level analysis. Data for heads of rovers and mutant sitters, fed and food deprived.

(a) ANOVA of total compound levels

| **Group** | **for F** | **for p** | **food F** | **food p** | **Int F** | **Int p** | **RNS** |
| --- | --- | --- | --- | --- | --- | --- | --- |
| **PS** | 405.0 | 3.88·10^-8^ | 164.1 | 1.31·10-6 | 39.2 | 2.43·10^-4^ | -0.34 |
| **TAG** | 5.20 | 0.052 | 73.1 | 2.70·10-5 | 5.59 | 0.046 | 0.36 |

Note: in all cases degrees of freedom (d.f.) for F statistics are 1,8.

(b) Group-level ANOVA

Polysaccharides (n=5 compounds). d.f. for all F values is 1,48

| **factor** | **F** | **p** |
| --- | --- | --- |
| **for** | 19.07 | 0.00005 |
| **food** | 7.33 | 0.0088 |
| **for x food** | 0.25 | 0.615 |
| **for x MW** | 4.41 | 0.040 |
| **food x MW** | 4.24 | 0.044 |
| **for x food x MW** | 4.77 | 0.033 |

Triaclyglycerols (n=13 compounds). d.f. for all F values is 1,136

| **factor** | **F** | **p** |
| --- | --- | --- |
| **for** | 44.99 | 3.91·10^-10^ |
| **food** | 408.47 | <1.0·10^-15^ |
| **for x food** | 66.58 | 1.33·10^-13^ |
| **for x MW** | 19.49 | 1.93·10^-5^ |
| **food x MW** | 3.45 | 0.065 |
| **for x food x MW** | 0.01 | 0.908 |

1. Total signal/noise (S/N) levels for compounds with MW consistent with polysaccharides (PS) and triacylglycerols (TAG) are summed within each replicate to give a total S/N for a metabolite group. Two-way Analysis of variance (ANOVA) was performed on these 12 observations (2 *for* x 2 food x 3 replicates). Interaction term (*for* x food) is significant for both PS and TAG.
2. Group-level analysis. Metabolites of different molecular weights respond differently to *for* and food. There is a significant interaction of MW with *for* in both PS and TAG. In PS, MW also interacts with food and with *for* x food. In other words, the direction of GEI depends on the polysaccharide MW. In TAG compounds, MW does not interact significantly with food or with *for* x food, but the interaction of *for* by food after accounting for MW is highly significant (p=1.33·10^-13^). See Supplementary Methods, below, for Group ANOVA details.
