# Supplementary material for: The Drosophila foraging Gene Mediates Adult Plasticity and Gene–Environment Interactions in Behaviour, Metabolites, and Gene Expression in Response to Food Deprivation
Source: PLoS Genet. 2009 Aug 21;5(8):e1000609. doi: 10.1371/journal.pgen.1000609 (PMC2720453; doi:10.1371/journal.pgen.1000609)
Supplement: Table S3 — Gene groups with significant GEI. (0.23 MB DOC) [file pgen.1000609.s006.doc]

# Supplementary Table 3. Gene groups with significant GEI.

Group-level ANOVA (Supplementary Methods) was calculated for all Gene Ontology (GO) gene groups in the Biological and Molecular categories. Only groups with at least 4 genes with mean expression above cutoff were considered. From these, we selected groups whose group-level GEI probability q(*for* x food) met a Storey-Tibshirani False Discovery Rate with q=0.05. Groups are ordered by the GEI level *I*, from most positive to most negative; that is, for those with large positive *I* rovers increase more when fed (or decrease more when food deprived (FD)) than sitters, while *I* negative implies rover genes decrease more when fed than sitters.

For clarity we place functional groups with similar roles in subsections of the table. Within a subsection such as Metabolic functional groups there is a transition from groups associated with protein translation activities which have positive *I* to those associated with carbohydrate, amino acid, and chitin catabolism and with mitochondrial oxidative phosphorylation which have negative *I* values.

Similarly, within the subsection Gene transcription and regulation, transcription and splicing have positive *I* while regulation has negative *I* values. Among signal transduction functional groups, only the growth-related cyclins show positive *I*. And most strikingly, among neuromuscular functional groups, all have negative *I*.

The effects of background (BG) differences between natural sitters and other strains were tested as described (Statistical Methods). The main effect of BG is significant for most groups, but GEI between BG and food are limited to functional groups associated with translation and transcription. Interestingly, none of the functional groups with significant negative *I* (*for* x food GxE) have significant BG x Food interaction, after FDR correction.

Note that although there are only slightly more gene groups with positive *I* levels than negative ones, there are many more groups with positive RNS (relative nutrient sensitivity) levels than negative, i.e. in most gene groups the magnitude of change for rovers is more than for sitters. The RNS values shown are for the rover-mutant sitter comparison; RNS for rover-natural sitters were highly correlated to those of rover-mutant sitter (Pearson *r* = 0.83, *t* = 12.91, df = 73, *p* < 2.2 10^-16^)

| **Gene Ontology group** | **N** | **q(*for*)** | **q(food)** | **q(for x food)** | **GEI *I*** | **q(BG)** | **q(BG x food)** | **RNS** |
| --- | --- | --- | --- | --- | --- | --- | --- | --- |
|  |  |  |  |  |  |  |  |  |
| **Miscellaneous:** |  |  |  |  |  |  |  |  |
| 6966 antifungal humoral response (sensu Protostomia) | **9** | 0.175 | **0.009** | ***0.013** | **0.359** | **0.014** | 0.777 | **0.617** |
| 48062 gravitactic behavior | **4** | 0.425 | 0.115 | ***0.030** | **0.321** | 0.131 | 0.371 | **0.475** |
|  |  |  |  |  |  |  |  |  |
| **Metabolic (anabolism/catabolism):** |  |  |  |  |  |  |  |  |
| 8320 protein carrier activity | **13** | 0.348 | **2.53 E-04** | *****5.14E-07** | **0.242** | **0.005** | 0.283 | **0.384** |
| 6334 nucleosome assembly | **8** | 0.343 | **0.007** | *****3.72E-05** | **0.233** | **4.14E-06** | 0.781 | **0.260** |
| 6616 SRP-dependent cotranslational protein-membrane targeting, translocation | **4** | 0.230 | **2.41 E-18** | ****0.0006** | **0.225** | **2.18E-06** | **6.73E-11** | **0.445** |
| 6606 protein-nucleus import | **17** | 0.140 | 0.235 | ***0.011** | **0.216** | 0.183 | 0.562 | **0.205** |
| 3747 translation release factor activity | **7** | 0.513 | 0.421 | ***0.013** | **0.196** | **0.003** | 0.333 | **0.158** |
| 339 RNA cap binding | **4** | 0.102 | 0.230 | ***0.009** | **0.181** | 0.193 | 0.757 | **0.123** |
| 5528 FK506 binding | **5** | 0.339 | **0.005** | ***0.039** | **0.169** | **0.007** | 0.293 | **0.227** |
| 4091 carboxylesterase activity | **14** | 0.069 | **0.033** | ***0.039** | **0.166** | **0.026** | 0.178 | **0.194** |
| 4239 methionyl aminopeptidase activity | **4** | 0.348 | **0.013** | ***0.035** | **0.158** | **0.008** | 0.586 | **0.265** |
| 8565 protein transporter activity | **23** | **0.021** | **1.73 E-05** | ***0.002** | **0.144** | **1.19E-04** | 0.253 | **0.217** |
| 8236 serine-type peptidase activity | **16** | 0.175 | **6.52 E-05** | ***0.010** | **0.139** | **0.005** | 0.235 | **0.192** |
| 4520 endodeoxyribonuclease activity | **6** | 0.348 | 0.390 | ***0.012** | **0.132** | **3.77E-04** | 0.283 | **0.024** |
| 4840 ubiquitin conjugating enzyme activity | **23** | 0.305 | 0.497 | ***0.014** | **0.103** | **0.003** | **4.18E-04** | **-0.125** |
| 30162 regulation of proteolysis and peptidolysis | **13** | 0.339 | 0.131 | ***0.029** | **0.087** | 0.227 | 0.134 | **0.016** |
| 4175 endopeptidase activity | **32** | 0.540 | **3.48 E-14** | ***0.008** | **0.072** | **2.96E-44** | **4.89E-21** | **0.110** |
| 6512 ubiquitin cycle | **45** | 0.212 | 0.417 | ***0.018** | **0.071** | 0.157 | 0.054 | **-0.081** |
| 6464 protein modification | **80** | 0.530 | 0.182 | ***0.038** | **0.066** | **2.14E-04** | 0.585 | **0.084** |
| 6486 protein amino acid glycosylation | **31** | 0.498 | **0.052** | ***0.020** | **0.064** | **4.41E-05** | 0.321 | **0.092** |
| 6412 protein biosynthesis | **175** | 0.459 | 0.327 | ***0.002** | **0.052** | **3.75E-30** | **8.75E-07** | **0.053** |
| 3735 structural constituent of ribosome | **158** | 0.259 | 0.353 | ***0.009** | **0.045** | **4.41E-35** | **1.08E-09** | **0.090** |
| 5975 carbohydrate metabolism | **98** | 0.058 | **3.59 E-05** | ***0.032** | **-0.076** | **0.006** | 0.474 | **0.118** |
| 6120 mitochondrial electron transport, NADH to ubiquinone | **28** | **1.26 E-09** | **4.47 E-12** | ***0.002** | **-0.077** | **2.22E-04** | 0.677 | **-0.163** |
| 15992 proton transport | **31** | 0.459 | **2.57 E-05** | ***0.028** | **-0.080** | **4.05E-12** | 0.455 | **-0.124** |
| 8137 NADH dehydrogenase (ubiquinone) activity | **23** | **1.65 E-08** | **1.22 E-08** | ***0.003** | **-0.084** | **8.81E-04** | 0.618 | **-0.184** |
| 9063 amino acid catabolism | **19** | **0.041** | **7.91 E-05** | ***0.024** | **-0.134** | 0.066 | 0.212 | **0.171** |
| 5977 glycogen metabolism | **8** | 0.401 | 0.462 | ***0.041** | **-0.176** | **4.92E-05** | 0.703 | **0.073** |
| 5041 low-density lipoprotein receptor activity | **6** | 0.313 | **0.035** | ***0.009** | **-0.177** | **0.002** | 0.639 | **0.265** |
| 15020 glucuronosyltransferase activity | **13** | 0.425 | 0.363 | ***0.029** | **-0.198** | **1.07E-06** | 0.593 | **0.153** |
| 6030 chitin metabolism | **15** | 0.514 | 0.460 | ***0.032** | **-0.258** | **7.63E-19** | 0.306 | **0.094** |
| 9072 aromatic amino acid family metabolism | **4** | 0.291 | **7.76 E-05** | ***0.014** | **-0.403** | **0.013** | 0.285 | **0.580** |
|  |  |  |  |  |  |  |  |  |
| **Gene transcription and regulation:** |  |  |  |  |  |  |  |  |
| 7307 chorion gene amplification | **5** | 0.426 | **0.027** | ***0.024** | **0.208** | 0.140 | 0.585 | **0.352** |
| 6268 DNA unwinding | **4** | 0.432 | 0.171 | ***0.005** | **0.192** | **5.99E-05** | 0.777 | **-0.140** |
| 6338 chromatin remodeling | **4** | 0.277 | 0.458 | ***0.005** | **0.162** | **3.54E-05** | 0.756 | **0.039** |
| 6289 nucleotide-excision repair | **6** | 0.513 | **0.020** | ***0.032** | **0.143** | 0.122 | 0.408 | **0.180** |
| 6325 establishment and/or maintenance of chromatin architecture | **11** | **1.82E-04** | **0.014** | ***0.016** | **0.141** | **0.013** | 0.488 | **-0.180** |
| 3899 DNA-directed RNA polymerase activity | **13** | 0.513 | 0.330 | ***0.005** | **0.119** | **0.004** | 0.283 | **0.113** |
| 3713 transcription coactivator activity | **15** | 0.508 | **0.003** | ***0.011** | **0.088** | **1.55E-06** | 0.698 | **0.162** |
| 7059 chromosome segregation | **22** | 0.459 | **0.024** | ***0.047** | **0.088** | 0.174 | 0.611 | **0.149** |
| 6281 DNA repair | **31** | 0.351 | **0.032** | ***0.024** | **0.067** | **2.71E-06** | 0.348 | **0.097** |
| 16251 general RNA polymerase II transcription factor activity | **45** | 0.383 | 0.390 | ***0.004** | **0.059** | **5.39E-13** | 0.722 | **0.025** |
| 6367 transcription initiation from RNA polymerase II promoter | **45** | 0.399 | 0.460 | ***0.005** | **0.054** | **3.21E-13** | 0.641 | **0.020** |
| 398 nuclear mRNA splicing, via spliceosome | **69** | 0.179 | **8.59E-07** | ***0.047** | **0.053** | **2.08E-13** | **0.012** | **0.104** |
| 3676 nucleic acid binding | **304** | 0.444 | 0.373 | ***0.003** | **0.038** | **3.80E-46** | **7.93E-06** | **0.038** |
| 6366 transcription from RNA polymerase II promoter | **78** | 0.466 | 0.474 | ***0.049** | **0.038** | **1.93E-04** | 0.258 | **0.035** |
| 7163 establishment and/or maintenance of cell polarity | **25** | **0.020** | 0.153 | ***0.017** | **-0.084** | **5.06E-06** | 0.768 | **0.074** |
| 7475 apposition of dorsal and ventral wing surfaces | **4** | 0.537 | 0.437 | ***0.049** | **-0.117** | 0.132 | 0.372 | **0.103** |
| 35151 regulation of tracheal tube size | **6** | 0.250 | **0.071** | ***0.004** | **-0.171** | **5.18E-12** | 0.756 | **-0.160** |
| 7498 mesoderm development | **81** | 0.417 | **0.046** | ***0.005** | **-0.081** | **2.82E-05** | 0.142 | **0.107** |
|  |  |  |  |  |  |  |  |  |
| **Signal transduction:** |  |  |  |  |  |  |  |  |
| 16538 cyclin-dependent protein kinase regulator activity | **9** | 0.522 | **0.001** | ***0.005** | **0.185** | **5.94E-05** | 0.450 | **0.303** |
| 7186 G-protein coupled receptor protein signaling pathway | **107** | 0.243 | **0.007** | ***0.032** | **-0.041** | **0.004** | 0.238 | **0.052** |
| 7242 intracellular signaling cascade | **121** | 0.053 | **5.44E-10** | ***0.044** | **-0.042** | 0.233 | 0.495 | **0.065** |
| 7165 signal transduction | **201** | **1.03E-04** | **2.02E-04** | ***0.004** | **-0.042** | **0.003** | 0.236 | **0.061** |
| 4715 non-membrane spanning protein tyrosine kinase activity | **8** | **1.03E-04** | **4.35E-04** | ***0.032** | **-0.096** | **0.001** | 0.300 | **0.131** |
| 16311 dephosphorylation | **13** | 0.178 | **0.038** | ***0.038** | **-0.102** | 0.082 | 0.512 | **0.162** |
|  |  |  |  |  |  |  |  |  |
| **Neuromuscular:** |  |  |  |  |  |  |  |  |
| 19226 transmission of nerve impulse | **64** | 0.457 | **0.008** | ***0.023** | **-0.059** | **0.047** | 0.556 | **0.098** |
| 7274 neuromuscular synaptic transmission | **15** | 0.277 | 0.539 | ***0.049** | **-0.076** | **4.37E-05** | 0.770 | **-0.005** |
| 5509 calcium ion binding | **112** | **0.046** | 0.380 | ***0.020** | **-0.090** | **8.72E-06** | 0.090 | **0.098** |
| 6814 sodium ion transport | **17** | 0.498 | 0.483 | ***0.030** | **-0.097** | **4.85E-09** | 0.258 | **0.082** |
| 4889 nicotinic acetylcholine-activated cation-selective channel activity | **8** | 0.057 | 0.380 | ***0.036** | **-0.102** | **2.73E-04** | 0.734 | **0.059** |
| 5267 potassium channel activity | **4** | 0.475 | 0.535 | ***0.041** | **-0.114** | **0.022** | 0.535 | **0.038** |
| 7270 nerve-nerve synaptic transmission | **18** | 0.228 | 0.446 | *****1.07E-05** | **-0.128** | **1.44E-07** | 0.671 | **0.008** |
| 8092 cytoskeletal protein binding | **22** | 0.198 | 0.436 | ***0.012** | **-0.141** | **1.32E-11** | 0.369 | **0.105** |
| 16079 synaptic vesicle exocytosis | **14** | 0.339 | **0.025** | ***0.003** | **-0.153** | **0.004** | 0.244 | **0.226** |
| 4890 GABA-A receptor activity | **5** | **0.005** | 0.531 | ***0.011** | **-0.154** | 0.105 | 0.441 | **-0.065** |
| 15276 ligand-gated ion channel activity | **4** | 0.493 | 0.362 | ***0.011** | **-0.175** | **0.004** | 0.747 | **0.056** |
| 8307 structural constituent of muscle | **8** | 0.407 | 0.350 | ***0.007** | **-0.363** | **1.77E-05** | 0.585 | **0.247** |
