# Supplementary material for: The Drosophila foraging Gene Mediates Adult Plasticity and Gene–Environment Interactions in Behaviour, Metabolites, and Gene Expression in Response to Food Deprivation
Source: PLoS Genet. 2009 Aug 21;5(8):e1000609. doi: 10.1371/journal.pgen.1000609 (PMC2720453; doi:10.1371/journal.pgen.1000609)
Supplement: Table S4 — Relative Nutrient Sensitivity (RNS) for metabolites. (0.06 MB DOC) [file pgen.1000609.s007.doc]

# Supplementary Table 4. Relative Nutrient Sensitivity (RNS) for metabolites

| **Mode** | **Description** | **RNS > 0** | **N** | **χ^2^** | **p** |
| --- | --- | --- | --- | --- | --- |
| 1101 | Positive ion electrospray, highly polar, not soluble in organic solvents at pH<2; quaternary amine, peptide, etc. | **42%** | 74 | 1.63 | 0.20 |
| 1102 | Negative ion electrospray, highly polar, not soluble in organic solvents at pH<2; sugar, phosphate, etc. | **34%** | 93 | 8.43 | 0.0037 |
| 1201 | Positive ion electrospray, non-polar neutral, soluble in organic solvents at pH<2; semi-volatile alcohol, ketone, etc | **68%** | 207 | 26.45 | 2.7·10^-7^ |
| 1202 | Negative ion electrospray; non-polar neutral and organic acids soluble in organic solvents at pH<2; organic or fatty acid, etc. | **88%** | 68 | 38.25 | 6.2·10^-10^ |
| 1203 | Positive ion atmospheric pressure chemical ionization, non-polar neutral soluble in organic solvents at pH<2; less volatile alcohol, ketone, etc. | **92%** | 166 | 116.39 | 3.9·10^-27^ |
| 1204 | Negative ion atmospheric pressure chemical ionization, organic acid soluble in organic solvents at pH<2; organic acid, etc. | **77%** | 150 | 41.61 | 1.1·10^-10^ |

Metabolite signal/noise ratio data detected using solvent 6 modes of FTICR MS (Fourier Transform Ion Cyclotron Resonance Mass Spectroscopy).  RNS was calculated (Methods) for each compound found in each of 6 modes. The number N of compounds found in each mode and the percentage of these with positive RNS (rovers change more than sitters) is given. This was tested against the null hypothesis that 50% of compounds would have RNS > 0 using a chi-square test. Compounds for 5 of the 6 detection modes different significantly from random RNS distributions. Only mode 1102, which detects compounds such as sugars, phosphates, etc. had significant excess of negative RNS (red, sitters change more than rovers), while the 4 modes 1201-1204 detecting compounds soluble in organic solvents (below black bar) all showed highly significant excess of positive RNS values (blue). In other words, the abundance of organic-solvent soluble compounds changed more strongly in rovers than in sitters in response to changes in food, while polar compounds such as polysaccharides changed more in sitters. However, polar compounds such as quaternary amines and peptides did not show significant RNS bias.

# 
