# Supplementary material for: The Drosophila foraging Gene Mediates Adult Plasticity and Gene–Environment Interactions in Behaviour, Metabolites, and Gene Expression in Response to Food Deprivation
Source: PLoS Genet. 2009 Aug 21;5(8):e1000609. doi: 10.1371/journal.pgen.1000609 (PMC2720453; doi:10.1371/journal.pgen.1000609)
Supplement: Table S5 — Complementation cross analysis of variance for insulin mutants. (0.09 MB DOC) [file pgen.1000609.s008.doc]

# Supplementary Table 5. Complementation cross analysis of variance for insulin mutants

This table presents ANOVA analysis for each of 3 quantitative complementation crosses between *for* strains and insulin pathway mutants. See Figure 6 for a graphical presentation of this data for the mutants *InR^93Dj-4^* and *Dp110^B^* in two genes which encode positive regulators of insulin signaling.

As described in Statistical Methods, a mixed-model ANOVA is performed with Day as a random factor. Hence each fixed effect term is tested against its interaction with Day. If the interaction of background with mutant gene is significant, the reduced comparison rover vs. sitter mutant, with no background difference, is used for testing the *foraging x mutant* interaction. Otherwise, *the foraging x mutant* interaction is tested in a mixed model using crosses with all three strains (rover, sitter and sitter mutant).

Factor names: *for* = *foraging* type (rover or sitter); BG=background, accounts for background differences between natural sitters and other strains); *gene* (one of *InR, Dp110,* or *foxo*), levels are mutant insulin gene or control balancer; Day= Day of testing.

**a) *for x InR^93Dj-4^*.**  The full model found interaction between BG and *InR* (F_1,10_=19.9, p=.002) so the reduced same-background rover vs sitter mutant comparison is shown

|  | *df* | *MS* | *df x Date* | *MS x Date* | *F* | *p* |
| --- | --- | --- | --- | --- | --- | --- |
| *for* | 1 | 0.086 | 10 | 0.0156 | 5.51 | **0.041** |
| *InR* | 1 | 0.346 | 10 | 0.0025 | 137.0 | **3.7** · 10^-7^ |
| ***for x InR*** | 1 | 0.073 | 10 | 0.0078 | 9.36 | **0.012** |
| *Date* | 10 | 0.019 |  |  | 2.87 | **0.021** |
| *Residual* | 20 | 0.007 |  |  |  |  |

**b) *for x Dp110^B^*.** Background interaction with *Dp110* is not significant (F_1,10_=1.22, p=.297) so we show the 3-strain comparison.

|  | *df* | *MS* | *df x Date* | *MS x Date* | *F* | *p* |
| --- | --- | --- | --- | --- | --- | --- |
| *for* | 1 | 0.186 | 10 | 0.0091 | 20.39 | **0.001** |
| *Dp110* | 1 | 0.388 | 10 | 0.0109 | 3.54 | 0.093 |
| *BG* | 1 | 0.031 | 10 | 0.0122 | 2.51 | 0.144 |
| ***for x Dp110*** | 1 | 0.069 | 10 | 0.0129 | 5.35 | **0.046** |
| *Date* | 10 | 0.027 |  |  | 4.25 | **0.0005** |
| *Residual* | 40 | 0.006 |  |  |  |  |

# c) *for x foxo^21^*. Background interaction is not significant (p=.646) so we show the 3-strain ANOVA. The *for x foxo* for the *for^R^ vs. for^s2^* comparison has *F_1,12_=4.45, p=0.07*.

|  | *df* | *MS* | *df x Date* | *MS x Date* | *F* | *p* |
| --- | --- | --- | --- | --- | --- | --- |
| *for* | 1 | 0.269 | 12 | 0.0164 | 16.39 | **0.0016** |
| *foxo* | 1 | 0.001 | 12 | 0.0170 | 0.037 | 0.851 |
| *BG* | 1 | 0.047 | 12 | 0.0114 | 4.08 | 0.065 |
| ***for x foxo*** | 1 | 0.040 | 12 | 0.0135 | 2.92 | 0.126 |
| *Date* | 12 | 0.041 |  |  | 6.61 | **4.6** · 10^-9^ |
| *Residual* | 125 | 0.007 |  |  |  |  |
