# Supplementary material for: The Drosophila foraging Gene Mediates Adult Plasticity and Gene–Environment Interactions in Behaviour, Metabolites, and Gene Expression in Response to Food Deprivation
Source: PLoS Genet. 2009 Aug 21;5(8):e1000609. doi: 10.1371/journal.pgen.1000609 (PMC2720453; doi:10.1371/journal.pgen.1000609)
Supplement: Table S6 — Meta-analysis: genes that respond to insulin differ between rovers and sitters. (0.14 MB DOC) [file pgen.1000609.s009.doc]

# Supplementary Table 6. Meta-analysis: Genes that respond to insulin differ between rovers and sitters.

Genes from studies negatively regulating levels of insulin-related pathway signaling by three distinct means (a - Gershman *et al.* 2007 overexpression of constitutively active *foxo* [29], b - Guertin *et al.* 2006 rapamycin [30], or c - Buch *et al.* 2008 ablation of *dilp3* expressing cells [28]) are compared to the rover-mutant sitter expression in our microarray data (a bioinformatic meta-analysis). See Figure 7 for graphical representation and overview. From genes showing significant up- or down-regulation in response to insulin signaling reduction, we selected those with expression levels in our arrays above cutoff. For each such gene we calculated the log_2_ fold change between rovers and mutant sitters (which share the same genetic background as rovers) for Fed and for FD flies. This results in 12 sets of measurements (12 = 3 studies x 2 (up/down regulated sets/study) x 2 (Fed or FD rover-sitter comparisons). Selection criteria for each study:

1. Genes from Cluster 1 and Cluster 2 of Gershman *et al*. from their Supp. Table 1
2. Genes from Guertin *et al.* Supplemental Affymetrix array data with fold change > 0.7 or <-0.7, p<0.01 at two hours post rapamycin
3. Genes from Buch *et al.* as deposited in GEO: GSE10625, with fold change >1.3 or <-1.3

If the same genes were affected by insulin in each study, our comparisons would not be independent. There was small overlap between the three studies’ gene sets, perhaps reflecting the different tissues used (*Drosophila* S2 cells, Kc cells, or adult bodies) and the different manipulations (in particular Guertin *et al.* used rapamycin, which reduces Tor signaling; Tor is downstream of *InR*-mediated signaling but is by no means the only *InR* target). To ensure full statistical independence, we removed from the Gershman *et al.* gene set any genes found in the other two studies, and similarly removed from the Guertin *et al.* set genes in the Buch *et al.* study (these removals did not change the significance or direction of within-study comparisons, and ensure that between study comparisons are independent). All genes affected by insulin in the 3 studies thus appear exactly once in these tests.

Once the set of genes up- or down-regulated for each study was determined, the rover-sitter log fold-change can be analysed for that set. We give the results of two non-parametric tests, the Kolmogorov-Smirnov test and the Wilcoxon rank sum test. In each case we test whether the selected gene set differs from the set of all genes with same mean expression (e.g. a two-sided test). The Kolmogorov-Smirnov and Wilcoxon tests are more robust to outlier values than parametric tests which assume normality, but less powerful; these are therefore conservative tests. In general Welch t-tests gave similar results (data not shown). In all comparisons the null hypothesis set of genes (all those in our arrays with expression greater than cutoff) has *n* = 5,944, so only *n* for the selected gene set is shown.

We performed the same non-parametric analysis on the GEI coefficient *I* from same-background and all-strain ANOVAs for selected gene to determine whether the GEI for genes affected by insulin differed from zero (*I_2_*, and again for *I* from the merged 3-strain set (*I_3_*).

We show results for non-overlapping gene sets from the three studies and for a pooled set (with no duplicates). Blue indicates significant positive values, red significant negative values. Genes with higher expression when insulin signaling is reduced have ***I*<0**; genes downregulated have ***I*>0.**

| **Study** | **insulin signals reduced by:** | **genes up- or down-regulated** | **Fed**, food deprived (**FD**), or Interaction ***I*** | ***n*** | **Kolmogorov-Smirnov** ***p*** | **Wilcoxon L** | **Wilcoxon *p*** |
| --- | --- | --- | --- | --- | --- | --- | --- |
|  |  |  |  |  |  |  |  |
| **Gershman**  ***et al*** | ***foxo* overexpression** | **up** | **FD** | 161 | 0.00054 | **0.091** | 0.00005 |
|  |  | **up** | **Fed** | 161 | 0.63 | 0.017 | 0.42 |
|  |  | **up** | ***I_2_*** | 161 | 0.00078 | **-0.039** | 0.00044 |
|  |  | **up** | ***I_3_*** | 161 | 5.8E-10 | **-0.083** | 8.1E-10 |
|  |  |  |  |  |  |  |  |
|  |  | **down** | **FD** | 468 | 9.0E-08 | **-0.049** | 9.6E-05 |
|  |  | **down** | **Fed** | 468 | 8.5E-06 | **0.060** | 2.2E-06 |
|  |  | **down** | ***I_2_*** | 468 | 2.8E-13 | **0.054** | <2.0e-16 |
|  |  | **down** | ***I_3_*** | 468 | 1.7E-13 | **0.073** | <1.0e-16 |
|  |  |  |  |  |  |  |  |
| **Guertin *et al*** | **rapamycin** | **up** | **FD** | 69 | 0.0027 | **0.090** | 0.0039 |
|  |  | **up** | **Fed** | 69 | 0.038 | 0.054 | 0.050 |
|  |  | **up** | ***I_2_*** | 69 | 0.2047 | -0.016 | 0.29 |
|  |  | **up** | ***I_3_*** | 69 | 0.0011 | **-0.058** | 0.0019 |
|  |  |  |  |  |  |  |  |
|  |  | **down** | **FD** | 74 | 0.94 | -0.007 | 0.81 |
|  |  | **down** | **Fed** | 74 | 0.025 | **0.092** | 0.0017 |
|  |  | **down** | ***I_2_*** | 74 | 0.013 | **0.049** | 0.0096 |
|  |  | **down** | ***I_3_*** | 74 | 0.0015 | **0.064** | 0.0016 |
|  |  |  |  |  |  |  |  |
| **Buch *et al*** | ***dilp3* knockdown** | **up** | **FD** | 29 | 8.1E-09 | **0.43** | 1.9E-06 |
|  |  | **up** | **Fed** | 29 | 0.00180 | **-0.14** | 0.019 |
|  |  | **up** | ***I_2_*** | 29 | 7.7E-13 | **-0.37** | 2.7E-07 |
|  |  | **up** | ***I_3_*** | 29 | 1.8E-07 | **-0.22** | 3.0E-05 |
|  |  |  |  |  |  |  |  |
|  |  | **down** | **FD** | 12 | 0.12400 | -0.080 | 0.474 |
|  |  | **down** | **Fed** | 12 | 0.00800 | **0.330** | 0.023 |
|  |  | **down** | ***I_2_*** | 12 | 0.00098 | 0.260 | 0.067 |
|  |  | **down** | ***I_3_*** | 12 | 0.03600 | -0.110 | 0.472 |
|  |  |  |  |  |  |  |  |
| **pooled** |  | **up** | **FD** | 228 | 3.0E-06 | **0.112** | 1.3E-08 |
|  |  | **up** | **Fed** | 228 | 0.35 | 0.016 | 3.72E-01 |
|  |  | **up** | ***I_2_*** | 228 | 1.1E-05 | **-0.047** | 9.0E-07 |
|  |  | **up** | ***I_3_*** | 228 | 5.7E-13 | **-0.087** | 1.5E-13 |
|  |  |  |  |  |  |  |  |
|  |  | **down** | **FD** | 553 | 1.5E-06 | **-0.043** | 0.00033 |
|  |  | **down** | **Fed** | 553 | 7.8E-07 | **0.068** | 3.42E-08 |
|  |  | **down** | ***I_2_*** | 553 | 1.6E-14 | **0.055** | <2.0e-16 |
|  |  | **down** | ***I_3_*** | 553 | 1.2E-13 | **0.071** | <1.0e-16 |
