# Supplementary material for: The Drosophila foraging Gene Mediates Adult Plasticity and Gene–Environment Interactions in Behaviour, Metabolites, and Gene Expression in Response to Food Deprivation
Source: PLoS Genet. 2009 Aug 21;5(8):e1000609. doi: 10.1371/journal.pgen.1000609 (PMC2720453; doi:10.1371/journal.pgen.1000609)
Supplement: Text S1 — Supplementary methods. (0.05 MB DOC) [file pgen.1000609.s010.doc]

# Supplementary Methods

*Group-level ANOVA*

ANOVA was performed on the log_2_ transformed data for a group of metabolites or genes, with *for,* food, compound (C) and molecular weight (MW) as fixed factors for metabolites, and *for,* food, and gene (G) as fixed factors for gene groups. Only genes in the top 42.5% by mean expression level or metabolites in top 50% were included in the analysis. (The same cutoffs are applied to individual gene ANOVA results). Group-level ANOVA detects trends over a group of compounds or genes hypothesized to behave similarly. Use of the compound factor C or gene factor G is equivalent to normalizing all the observations for a single compound or gene to have mean of zero and thus correctly accounts for degrees of freedom due to this normalization. We do not show F and p values for C or G as these are only formal normalizing factors, not testable for significance.

Significance of *for,* food, or *for* x food terms in the group-level ANOVA implies there is a significant common, group-level effect of that factor on all genes or compounds in the group. Individual genes or compounds may vary around the group trend, but if the group factor is significant it is a more parsimonious way of explaining variation in the group than by fitting ANOVA models to each gene or compound separately.

Where there are additional attributes of compounds or genes, such as MW, which are hypothesized to affect the data, these attributes can be added to the group-level ANOVA as covariates. We illustrate this with compounds in Supplementary Table 2, where MW is the additional covariate.
